# Identification of key biomarkers and immune infiltration in systemic lupus erythematosus by integrated bioinformatics analysis

**DOI:** 10.1186/s12967-020-02698-x

**Published:** 2021-01-19

**Authors:** Xingwang Zhao, Longlong Zhang, Juan Wang, Min Zhang, Zhiqiang Song, Bing Ni, Yi You

**Affiliations:** 1 Department of Dermatology, Southwest Hospital, Army Medical University, (Third Military Medical University), Chongqing, 400038 China; 2 Department of Pathophysiology, College of High Altitude Military Medicine, Army Medical University, (Third Military Medical University), Chongqing, China; 3 State Key Laboratory of Genetic Resources and Evolution, Kunming Institute of Zoology, Chinese Academy of Sciences, Kunming, 650223 China; 4 Kunming College of Life Science, University of Chinese Academy of Sciences, Kunming, 650204 China

**Keywords:** Systemic lupus erythematosus, Immune infiltration, Integrated bioinformatics, IFI27, Biomarkers

## Abstract

**Background:**

Systemic lupus erythematosus (SLE) is a multisystemic, chronic inflammatory disease characterized by destructive systemic organ involvement, which could cause the decreased functional capacity, increased morbidity and mortality. Previous studies show that SLE is characterized by autoimmune, inflammatory processes, and tissue destruction. Some seriously-ill patients could develop into lupus nephritis. However, the cause and underlying molecular events of SLE needs to be further resolved.

**Methods:**

The expression profiles of GSE144390, GSE4588, GSE50772 and GSE81622 were downloaded from the Gene Expression Omnibus (GEO) database to obtain differentially expressed genes (DEGs) between SLE and healthy samples. The gene ontology (GO) and Kyoto Encyclopedia of Genes and Genomes (KEGG) pathway enrichments of DEGs were performed by metascape etc. online analyses. The protein–protein interaction (PPI) networks of the DEGs were constructed by GENEMANIA software. We performed Gene Set Enrichment Analysis (GSEA) to further understand the functions of the hub gene, Weighted gene co‐expression network analysis (WGCNA) would be utilized to build a gene co‐expression network, and the most significant module and hub genes was identified. CIBERSORT tools have facilitated the analysis of immune cell infiltration patterns of diseases. The receiver operating characteristic (ROC) analyses were conducted to explore the value of DEGs for SLE diagnosis.

**Results:**

In total, 6 DEGs (IFI27, IFI44, IFI44L, IFI6, EPSTI1 and OAS1) were screened, Biological functions analysis identified key related pathways, gene modules and co‐expression networks in SLE. IFI27 may be closely correlated with the occurrence of SLE. We found that an increased infiltration of moncytes, while NK cells resting infiltrated less may be related to the occurrence of SLE.

**Conclusion:**

IFI27 may be closely related pathogenesis of SLE, and represents a new candidate molecular marker of the occurrence and progression of SLE. Moreover immune cell infiltration plays important role in the progession of SLE.

## Background

Systemic lupus erythematosus (SLE) is the common autoimmune diseases in the world, which have influenced the adult involving multiple organs, mostly in young women, with an increasing number of early, mild and atypical cases [1]. SLE is the result of different pathogenesis, and has showed the different clinical manifestation and cellular and molecular foundation. The pathogenesis of SLE focuses on autoantibodies and immune complexes, inflammatory processe and tissue destruction [2–5]. However, the pathophysiologic mechanisms of SLE have not been investigated thoroughly. Therefore, it is very important to explore the molecular characteristics and mechanism of SLE occurrence, progression to provide new strategies for the effective prevention, diagnosis and treatment of SLE.

In recent studies, microarrays based on high-throughput platforms have widely used to explore and identify the promising biomarkers for diagnosis and prognosis of disease at the genome level. Numerous studies [6–8] have demonstrated that the pathophysiological process for the development of SLE are closely associated with the mutation and abnormal expression of genes, which include TNFSF4, NCF1-339, CXorf21, etc. A previous study demonstrated that IFI44L promoter methylation as a blood biomarker for systemic lupus erythematosus [9]. In an animal research, researchers demonstrated that there is the association between expression of IFIT1 in podocytes of MRL/lpr mice and the renal pathological changes it causes [10]. Wang et al. [11] showed that there was the association of abnormal elevations in IFIT3 with overactive cyclic GMP-AMP synthase**/**Stimulator of interferon genes signaling in human systemic lupus erythematosus monocytes. Moreover, It has been showed that cGAS activation causes lupus-like autoimmune disorders in a TREX1 mutant mouse model [12]. In addition, IRF5 risk variants associate with elevated IRF5 expression and IFN production in SLE blood cells [13]. Therefore, it is imperative to explore the accurate molecular targets included in occurrence and progression of SLE, in order to make a contribution to the diagnosis and treatment of SLE.

Herein, we analysed four mRNA microarray datasets from Gene Expression Omnibus (GEO) to screened differentially expressed genes (DEGs) between SLE and healthy samples. Subsequently, the molecular mechanisms of the pathogenesis of SLE were subsequently explored via enrichment analysis of functions and pathways. Protein–protein interaction (PPI) network analysis was carried out to explore relationships between DEGs, 6 hub genes were screened. IFI27 was identified as a key hub gene closely correlated with the progression of SLE. CIBERSORT was used to evaluate abundance of immune infiltrates. WGCNA and GSEA analysis was used to analyse the mechanism by which IFI27 may affect the pathogenesis of SLE. The findings provide new candidate molecular markers for studying the pathogenesis of SLE. Data processing was performed by using R software (Version 3.6.1; https://www.r-project.org/) and bioconductor packages (http://www.bioconductor.org/), together with the online website such as metascape etc. It is anticipated that the novel DEGs and pathways between SLE and healthy controls identified in this study may shed light on the underlying molecular.

## Materials and methods

### Access to GEO datasets

Genes were screened using the GEO (http://www.ncbi.nlm.nih.gov/geo) database [14]. GSE144390, GSE4588, GSE50772 and GSE81622, which all identify genes and pathways involved in the formation of SLE compare with normal individuals, were obtained from the GEO. The GSE50772 [15], GSE4588 series on the GPL570 platform (Affymetrix Human Genome U133 Plus 2.0 Array), the GSE144390 series on the GPL6244 platform (Affymetrix Human Gene 1.0 ST Array), and the GSE81622 series [16] on the GPL10558 platform (Illumina HumanHT-12 V4.0 expression beadchip), the basic information of the datasets selected is showed in Additional file 1: Table S1. The probes were transformed into the homologous gene symbol by means of the platform’s annotation information.

### DEGs identified by GEO2R

The GEO2R (http://www.ncbi.nlm.nih.gov/geo/geo2r), is an online data analysis tool, and was used to screen the DEGs between SLE and healthy controls. We established the six differential experimental groups for four GEO series, GSE4588 series divided into GSE4588 CD4 T cells and GSE4588 B cells series, GSE81622 divided into GSE81622 SLE and GSE81622 LN series, GEO2R could compare the differential classifications so that the DEGs would be identified. Genes without a corresponding gene symbol and genes with more than one probe set are separately removed, The values for statistical significance were set as adjusted p value ≤ 0.05 and |Fold change|≥ 1. In order to identify significant DEGs, the Venn online tool (http://bioinformatics.psb.ugent.be/webtools/Venn/) was used to draw a Venn map, and overlapping DEGs were retained for further analysis.

### Analyses of DEGs

Volcano maps were drawn using the volcano plotting tool (http://soft.sangerbox.com/). TBtools (http://www.tbtools.com/) was used to draw expression heatmap of DEGs in different series. The correlation analysis between gene–gene and series-series was used in tool (http://soft.sangerbox.com/).

### Functional annotation and pathway enrichment analysis

To functionally annotate DEGs identified by the aforementioned comparison groups, annotation and visualization of GO terms was used by GO enrichment analysis (http://enrich.shbio.com/index/ga.asp) and metascape (http://metascape.org/gp/index.html#/main/step1). The overlaps between differently expressed gene lists of GO terms are performed by enrichment analysis circle diagram (http://soft.sangerbox.com/). The DEGs were then introduced into the FunRich (functional enrichment analysis tool) (http://www.funrich.org/) for KEGG pathway analysis. GENEMANIA (http://genemania.org/search/) was used to construct a gene–gene interaction network for DEGs to evaluate the functions of these genes.

### Enrichment analysis by gene set enrichment analysis (GSEA)

GSEA version 4.1.0 software was used to analyze genes function from the GSEA website MSIGDB database (http://software.broadinstitute.org/gsea/msigdb) [17]. The default weighted enrichment method was applied for enrichment analysis. The random combination was set for 1000 times. GO and KEGG pathway enrichment analysis were performed for IFI27 high and low expression using GSEA analysis. FDR < 0.25, NOM p-value < 0.05 and |NES|> 1 were considered significant enrichment. Datas used were shown in Additional file 2: Table S2.

### Construction of weighted gene co-expression network analysis (WGCNA)

The WGCNA package in R was utilized to build a coexpression network targeting DEGs [18]. We established a weighted adjacency matrix, defined a correlation power (soft thresholding parameter) showing strong relations between genes and penalizing the weak correlation. Then we converted the adjacency into a topological overlap matrix (TOM) to measure the network connectivity of genes, and the TOM summed up the adjacent genes for the network gene ratio and calculated the corresponding dissimilarity. We used average linkage hierarchical clustering based on TOM dissimilarity measurement to classify genes showing similar expression profiles with gene modules, which were represented by branches and different colors of the cluster tree, constructed module relationships, calculation of the correlation between gene modules and phenotypes, and the modules related to clinical traits were identified. Datas used were shown in Additional file 3: Table S3. Scripts were shown in Additional file 4.

### Evaluation of immune cell infiltration

To evaluate abundance of immune infiltrates, We uploaded the gene expression matrix data to CIBERSORT (https://cibersort.stanford.edu/), [19] and obtained the immune cell infiltration matrix. Then, we used “corrplot” package [20] to draw a correlation heatmap to visualize the correlation of 22 types of infiltrating immune cells, “ggplot2” package [21] was used to perform PCA clustering analysis on immune cell infiltration matrix data to draw a two-dimensional PCA clustering map, and to draw violin diagrams to visualize the differences in immune cell infiltration. Datas used were shown in Additional file 5: Table S4, Additional file 6: Table S5, Datas of results were shown in Additional file 7: Table S6. Scripts were shown in Additional file 8.

### Principal component analysis (PCA)

The Pearson’s correlation test was performed to verify intra-group data repeatability in the per group. The R programming language was used to provide the software and operating environment for statistical analysis and drawing of graphs. The intra-group data repeatability of the dataset was tested by sample clustering analysis.

### ROC analysis

The multivariate modelling with combined selected genes were used to identify biomarkers with high sensitivity and specificity for SLE diagnosis by using visualization tool (https://hiplot.com.cn/basic/roc). Used one data as training and other as validation sample iteratively. The receiver operator characteristic curves were plotted and area under curve (AUC) was calculated separately to evaluate the performance of each model using the R packages “pROC” [22]. A AUC > 0.9 indicated that the model had a good fitting effect.

## Results

### Identification and analysis of DEGs in datasets

**Fig. 1:**
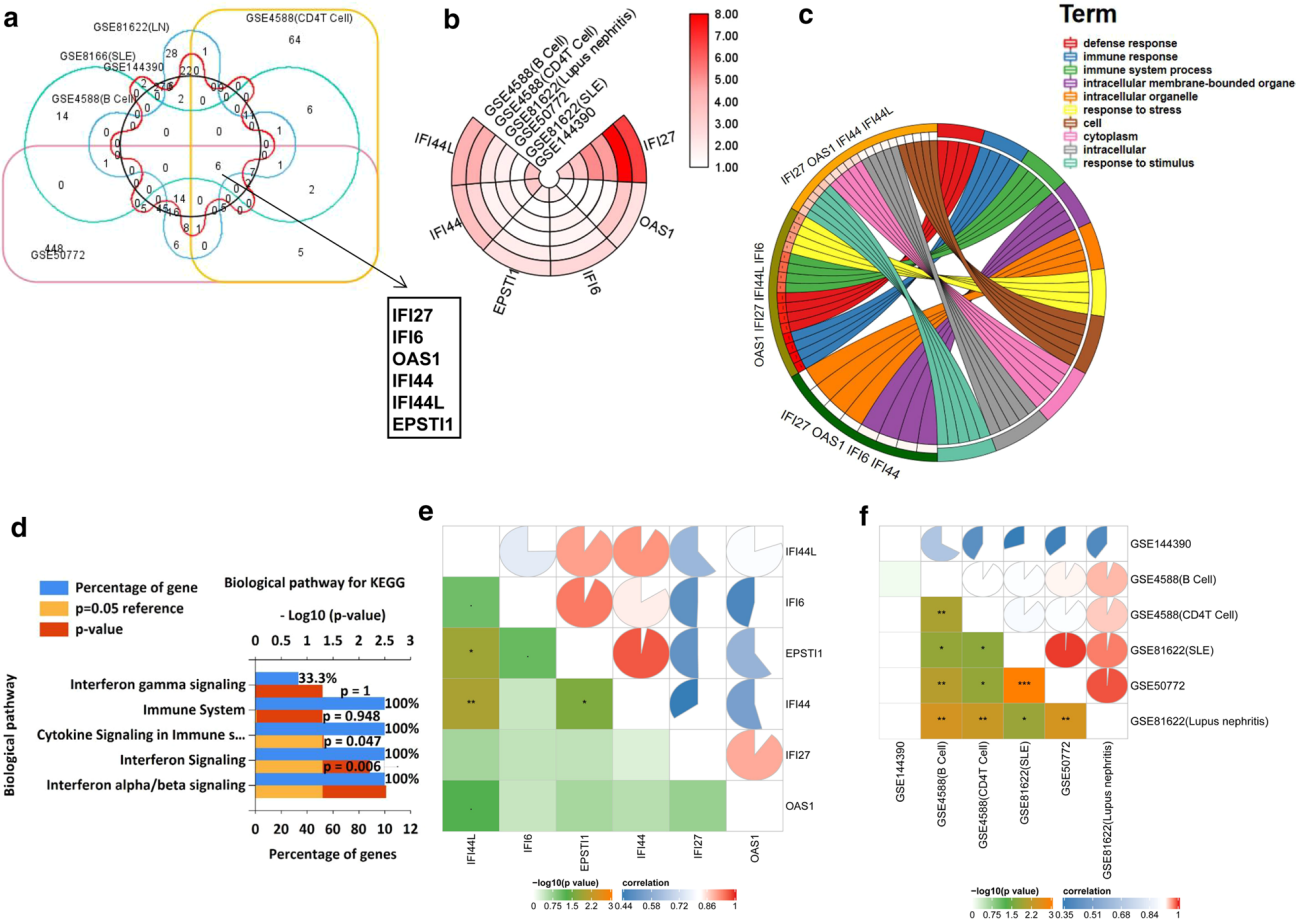
Gene expression, correlation and enrichment analysis, showing the significant function related to DEGs. **a** DEGs were identified from GSE81622 (SLE), GSE81622 (Lupus nephritis), GSE4588(CD4 T Cell), GSE4588(B Cell), GSE50772 and GSE144390 gene expression profiling datasets based on **|**fold change**|**≥ 1 and adjusted p value < 0.05. The six datasets share 6 overlapping DEGs. **b** Heatmap of DEGs derived from integrated analysis. Each circle represents one dataset and each sector represents one gene, the gradual color ranged from white to red represents the changing process of up-regulation. Up-regulated genes were marked in red, respectively. **c** GO term enrichment analysis of module genes. **d** Top 5 terms of KEGG analysis in biological pathway category (Ranged by p value). **e** Corrgrams were derived based on pearson value between DEGs, respectively. **f** Corrgrams were derived based on pearson value between six datasets, respectively. DEGs, differentially expressed genes; GO, Gene Ontology; KEGG, Kyoto Encyclopedia of Genes and Genomes

**Fig. 2:**
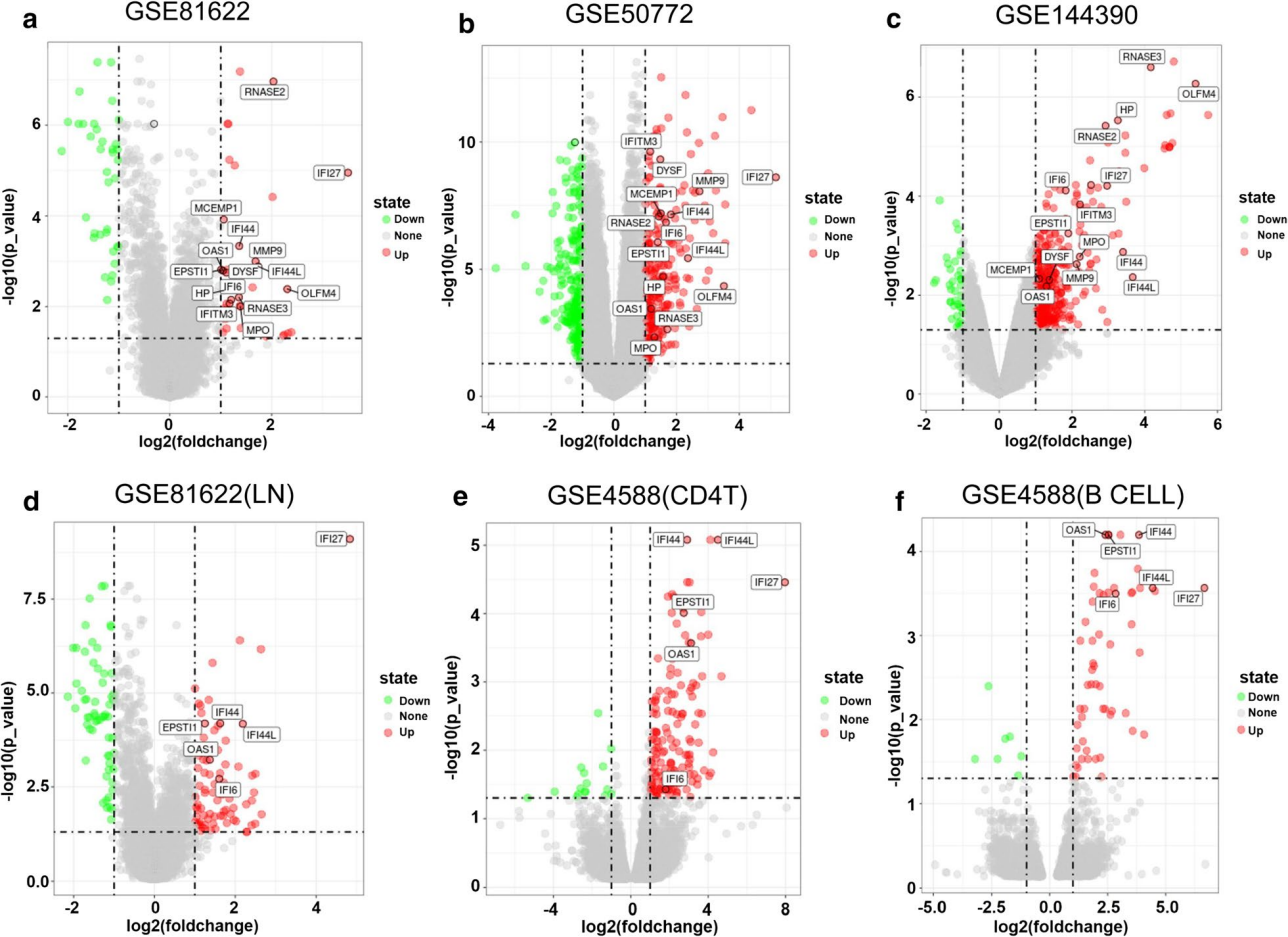
The volcano plot illustrates DEGs. The volcano plot illustrates DEGs between control and SLE after analysis of the **a** GSE81622(SLE), **b** GSE50772, **c** GSE144390, **d** GSE81622 (Lupus nephritis), **e** GSE4588(CD4 T Cell), **f** GSE4588(B Cell) dataset with GEO2R. DEGs, differentially expressed genes

Based on the high throughput analysis, DEGs in the six microarray datasets (GSE4588(CD4 T cells), GSE4588(B cells), GSE81622(SLE), GSE81622 (LN), GSE144390, GSE50772) were screened after the chip results were normalised (Additional file 9: Table S7). As shown in the Venn map, 6 genes overlapped in the six datasets (Fig. 1a). Based on the integration analysis, 6 significantly up-regulated genes were shown by heatmap (Fig. 1b). GO enrichment analysis was used to evaluate the potential mechanism of DEGs from molecular function, biological process, and cellular component categories. The results showed that these genes were functionally associated with several immune related biological processes. The circos present the overlap between differently expressed gene lists of six datasets at the shared term level (Fig. 1c). KEGG pathway analysis showed the related genes were involved in interferon signaling, interferon alpha/beta signaling pathways (Fig. 1d). The circos present the overlap between differently expressed gene lists of six datasets at the gene level was shown in the Additional file 10: Figure S1. The bubble chart present GO term of 6 DEGs from the six datasets was shown in the Additional file 10: Figure S2. Corrgrams were derived based on pearson value between DEGs (Fig. 1e, Additional file 11: Table S8). Corrgrams were derived based on pearson value between six datasets (Fig. 1f, Additional file 12: Table S9). We further investigated the difference between the expression levels of the six genes, The results showed that IFI27 is the significantly up-regulated gene in six datasets (Fig. 2a–f).

### Construction of a ceRNA network

**Fig. 3:**
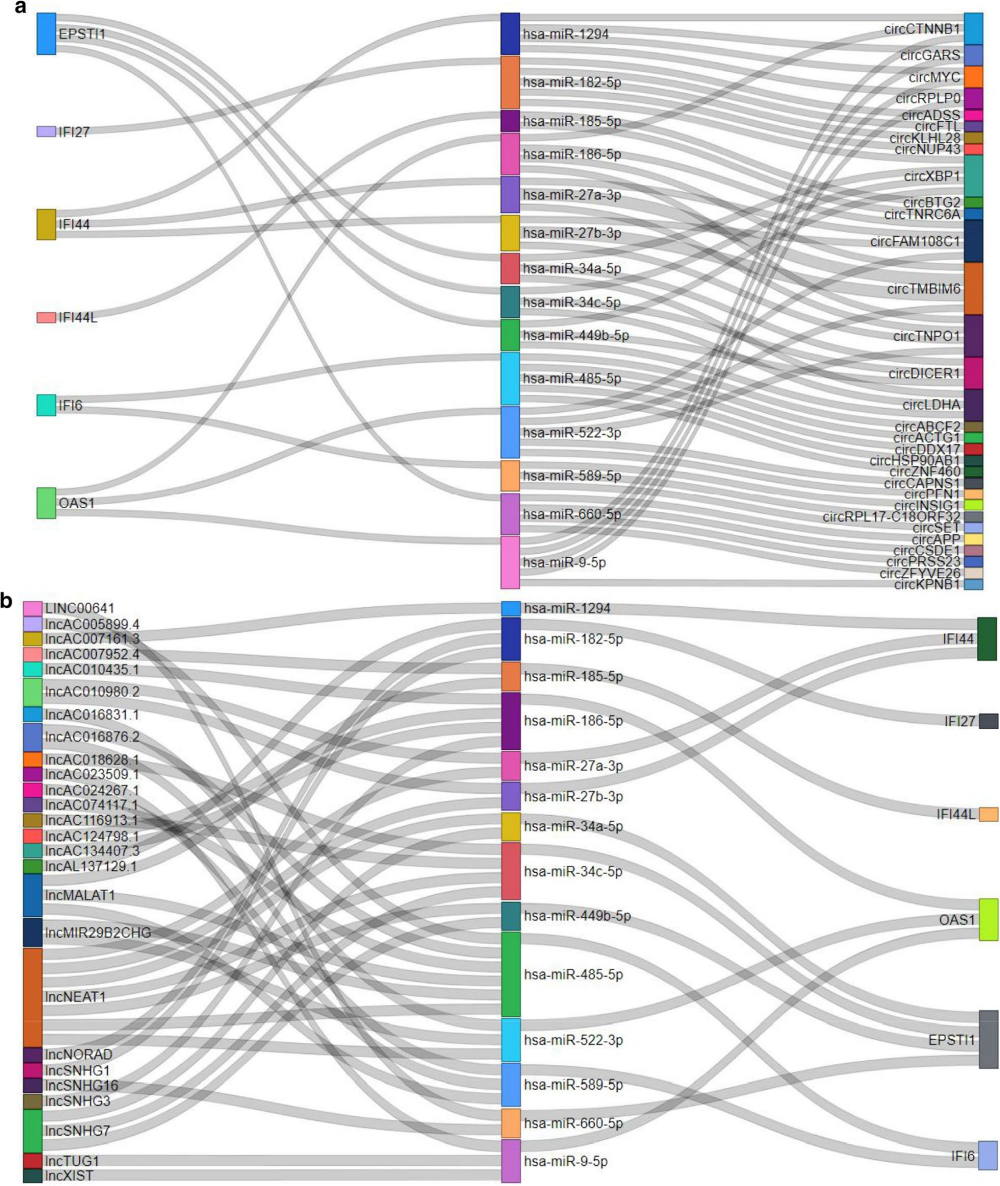
Sankey diagram for the ceRNA network about DEGs. **a** CirRNAs-miRNAs-mRNAs network, **b** LncRNAs-miRNAs-mRNAs network, each rectangle represents a gene, and the connection degree of each gene is visualized based on the size of the rectangle

To better understand the effect of circRNAs and lncRNAs on mRNAs mediated by combination with miRNAs, we built two ceRNA network based on the abovementioned data and used the Power BI (https://powerbi.microsoft.com/zh-cn/) to visualize the network (Fig. 3a, b). CircRNAs and lncRNAs interact with miRNAs retrieved from the TargetScan (http://www.targetscan.org) database. CircRNAs and lncRNAs with mircoRNAs have the weak interaction were removed, basing on clipExpNum from large-scale CLIP-Seq data of the TargetScan database (Additional file 13: Table S10, 11). Moreover, the miRNAs can interact with mRNAs more than two of seven the databases were chosen (Additional file 14: Table S12). Seven databases were PITA, RNA22, miRmap, microT, miRanda, PicTar, TargetScan.

### Functional enrichment analyses and PCA of datasets

**Fig. 4:**
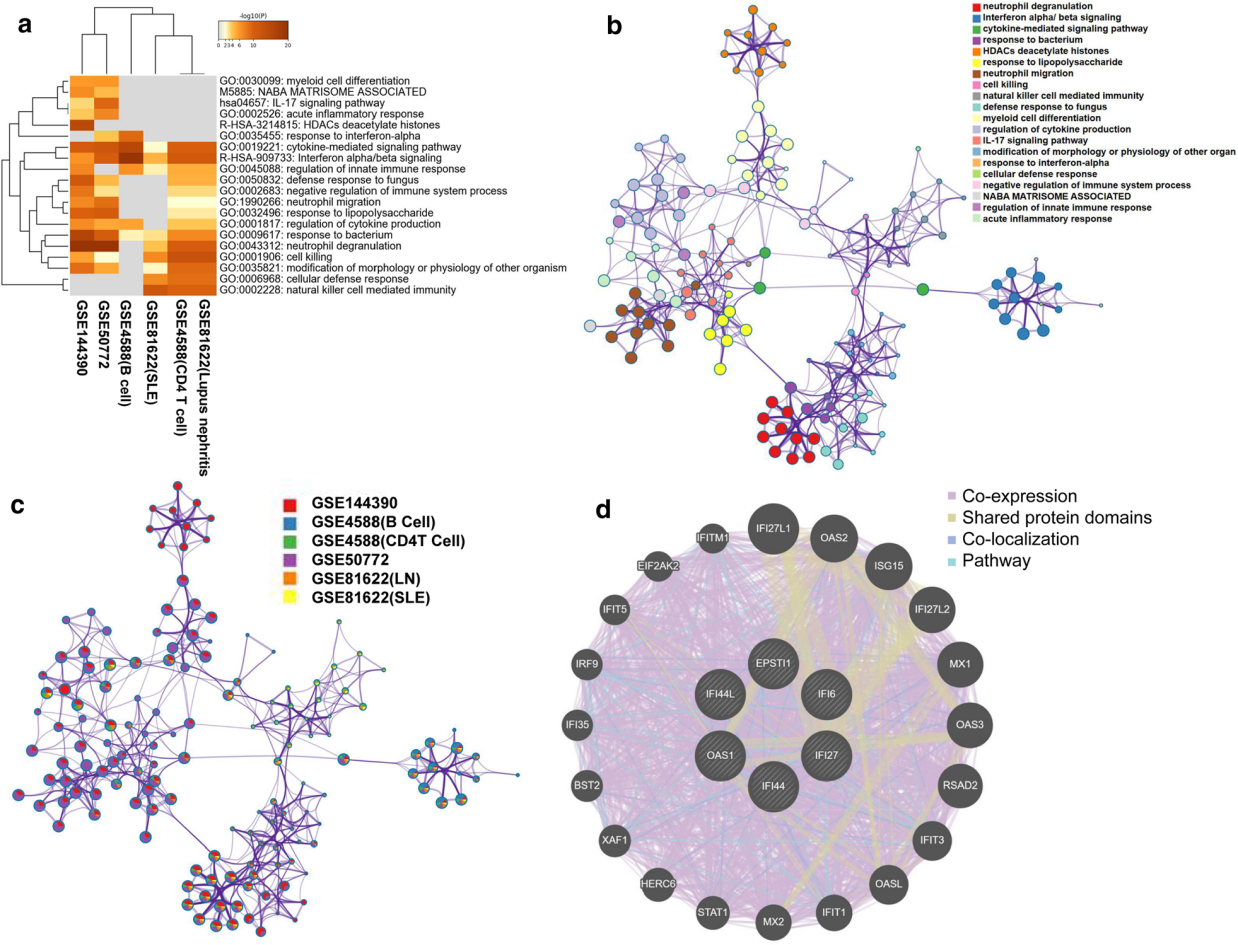
Detailed information relating to changes in the biological function of DEGs in datasets through the enrichment analyses. **a** Heatmap of enriched terms across input gene expressed matrix of six datasets, via the Metascape. **b** Network of enriched terms colored by cluster identity, where nodes that share the same cluster identity are typically close to each other. **c** Network of enriched terms and genes colored by datases, where terms containing more genes tend to have a more significant. **d** The gene–gene interaction network for DEGs was analyzed using the GeneMANIA database. The 20 most frequently changed neighboring genes are shown. Each node represents a gene. The node color represents the possible functions of the respective gene

The enrichment analysis of metascape results revealed that there were markedly enriched in cytokines-regulate signaling pathways, interferon alpha/beta signaling pathways, response to bacterium (Fig. 4a, b). What’s more, the enrichment analysis of metascape also demonstrates that the DEGs between control and SLE were markedly enriched in the six datasets (Fig. 4c). We constructed a gene–gene interaction network for DEGs to analyze the function of these genes using the GeneMANIA database. The hub node representing DEGs was surrounded by 20 nodes representing genes that were significantly correlated with DEGs (Fig. 4d).

**Fig. 5:**
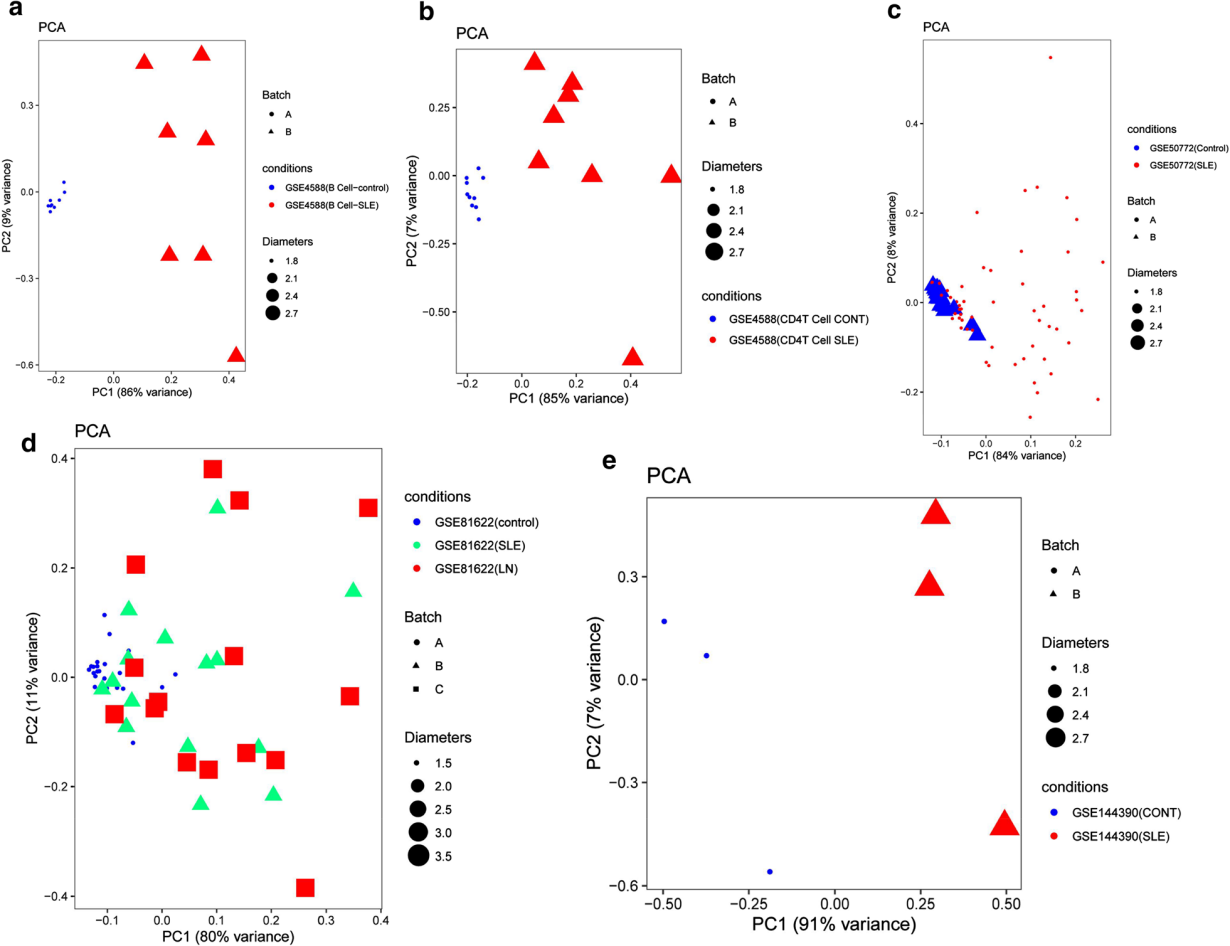
Principal components analyses performed on all datasets. **a** GSE4588 (B Cell), **b** GSE4588 (CD4T Cell). **c** GSE50772, (d) GSE81622, **e** GSE144390 datasets. Principal component 1 (PC1) and principal component 2 (PC2) are used as the X-axis and Y-axis, respectively, to draw the scatter diagram, where each point represents a sample. The farther the two samples are from each other, the greater the difference is between the two samples in gene expression patterns

To further validate the intra-group data repeatability, Principal component analysis (PCA) demonstrated a different distribution pattern between the SLE and control groups, based on the expression of genes in all samples. The distances between per samples in the control group were close, and distances between per samples in the SLE group were also close in the dimension of PC1 (Fig. 5a–e). This was indicative of the difference of two groups.

### Gene set enrichment analysis (GSEA) of IFI27-associated gene set

**Fig. 6:**
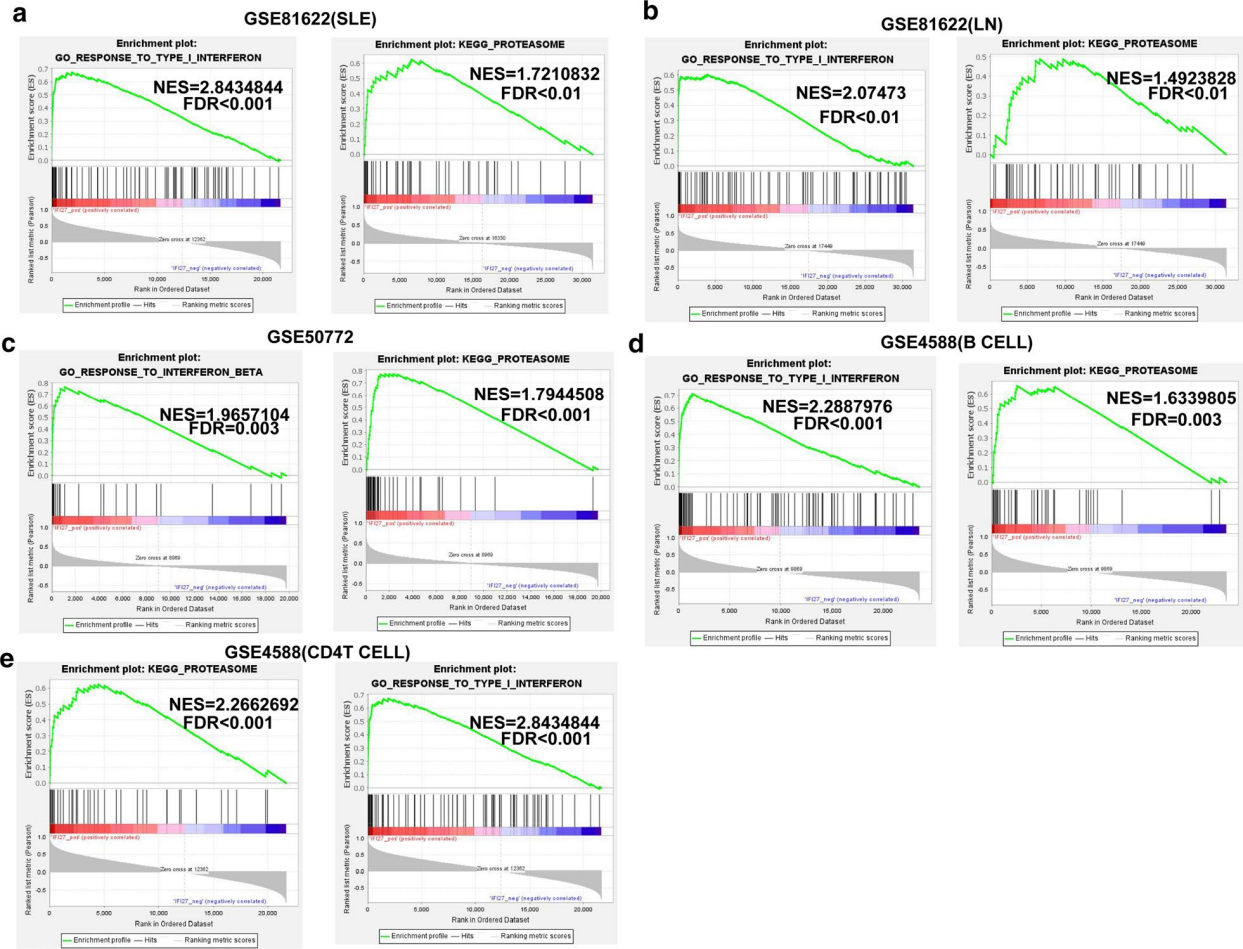
Gene set enrichment analysis (GSEA) was used to analyze the signaling pathways enrichment in different groups. GSEA used to validate the gene signatures of IFI27 in **a** GSE81622 (SLE), **b** GSE81622 (LN), **c** GSE50772, **d** GSE4588 (B cell), **e** GSE4588 (CD4 T cell), including response to type I interferon signaling and the protesome KEGG pathway. Normalized enrichment score (NES) indicated the analysis results across gene sets. False discovery rate (FDR) presented if a set was significantly enriched

We used GSEA to analyze enriched GO and KEGG pathways in the samples with the IFI27 highly expressed in different datasets. Then we screened out one commonly enriched pathway: Response to type I interferon signaling and the protesome KEGG pathway (Fig. 6a–f). Results of this study indicated that interferon response is one of the biological pathway most relevant to the pathogenesis of SLE.

### Construction of co-expression modules by weighted gene co-expression network analysis (WGCNA) of datasets

**Fig. 7:**
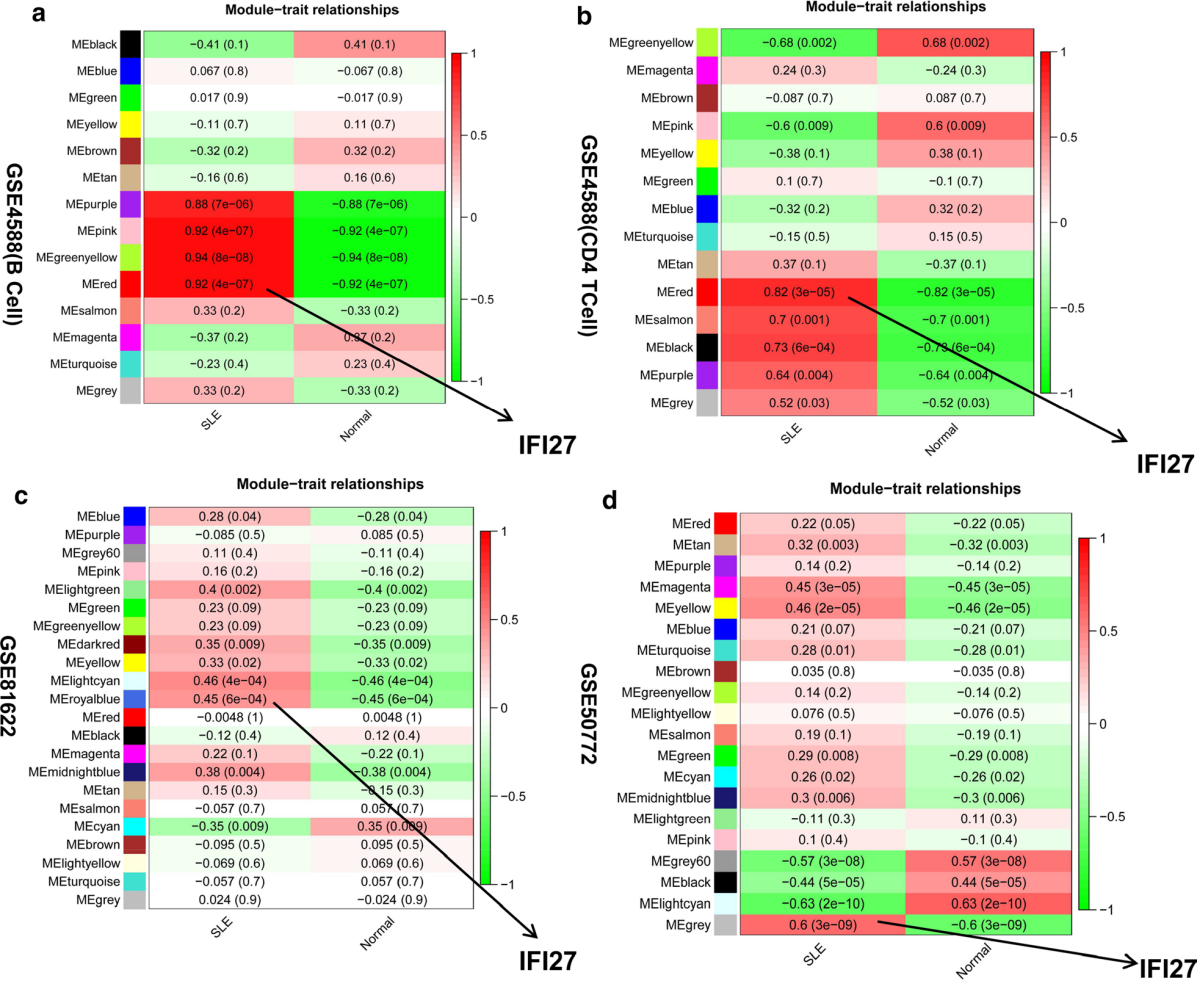
Identification of weighted gene co‑expression network modules associated with SLE in four datasets. **a** GSE4588(B Cell), **b** GSE4588(CD4 T Cell), **c** GSE81622, **d** GSE50772, The eigengene adjacency heatmap of the correlation between module genes and groups of SLE and control. Every color represents one co-expression module. IFI27 in the module of chiefly enriched in correlated with the occurence of SLE

In this study, we obtained the expression matrices of all samples in four dataset (GSE50772, GSE4588(B Cell), GSE4588(CD4 T Cell), GSE81622, Additional file 5: Table S4). Then we selected the top 30–50% variant genes (less than 5000) for co‐expression analysis (Additional file 3: Table S3). We excluded dataset of GSE144390 because of the small number of samples. The eigengene adjacency heatmap showed that the red module was the most positively correlated with occurrence of SLE, and the green module was the most negatively correlated with occurrence of SLE. Enrichment analysis performed in this study indicated that IFI27 in the module of chiefly enriched in correlated with the occurence of SLE (Fig. 7a–d, Additional file 15: Figure S3, 4, Additional file 16: Table S13).

### Immune cell infiltration results

**Fig. 8:**
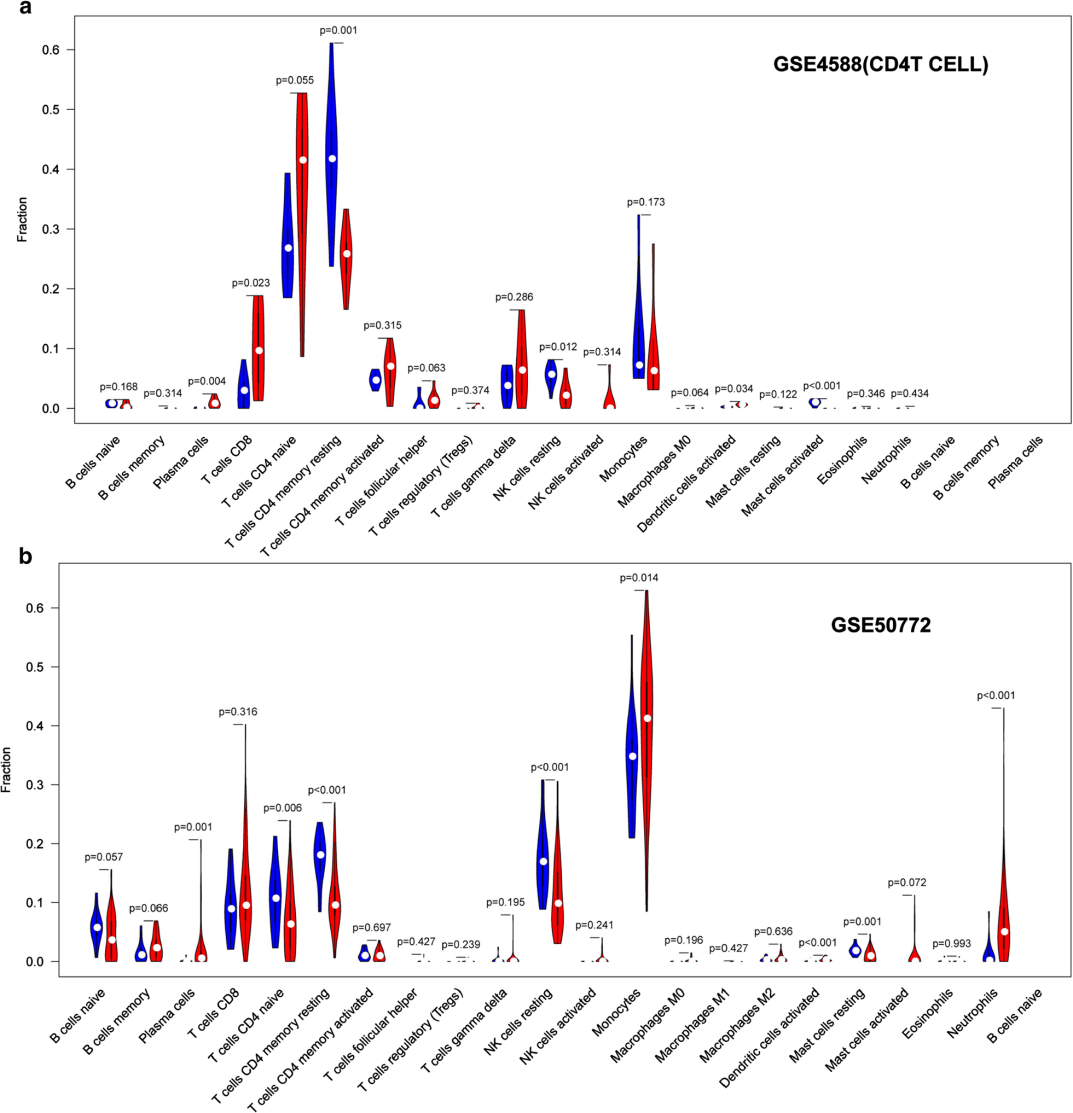
Violin diagram of the proportion of 22 types of immune cells. **a** GSE4588(CD4 T Cell), **b** GSE50772, showed the difference in infiltration between the two groups

**Fig. 9:**
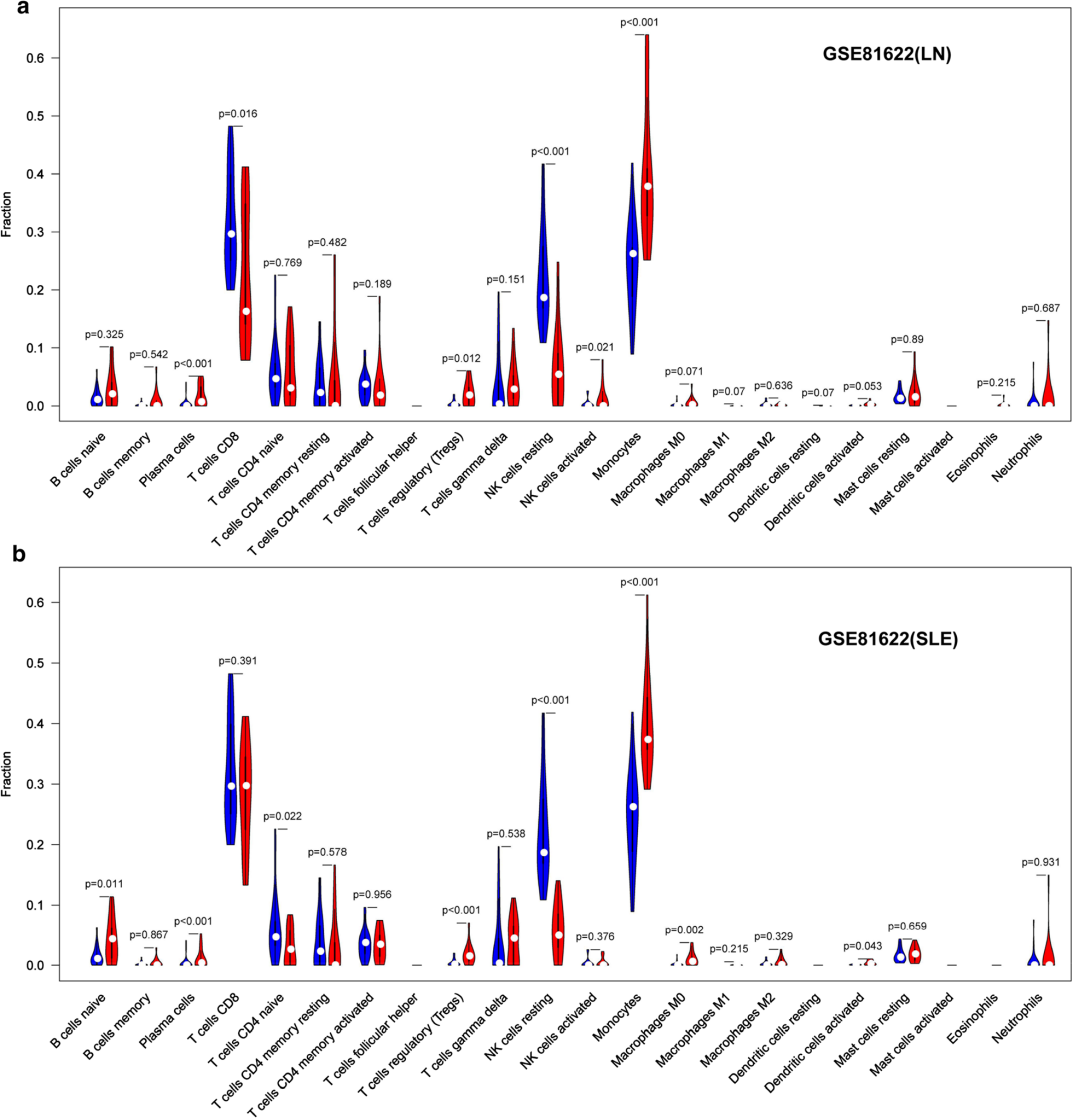
Violin diagram of the proportion of 22 types of immune cells. **a** GSE81622(LN), **b** GSE81622(SLE), showed the difference in infiltration between the two groups

**Fig. 10:**
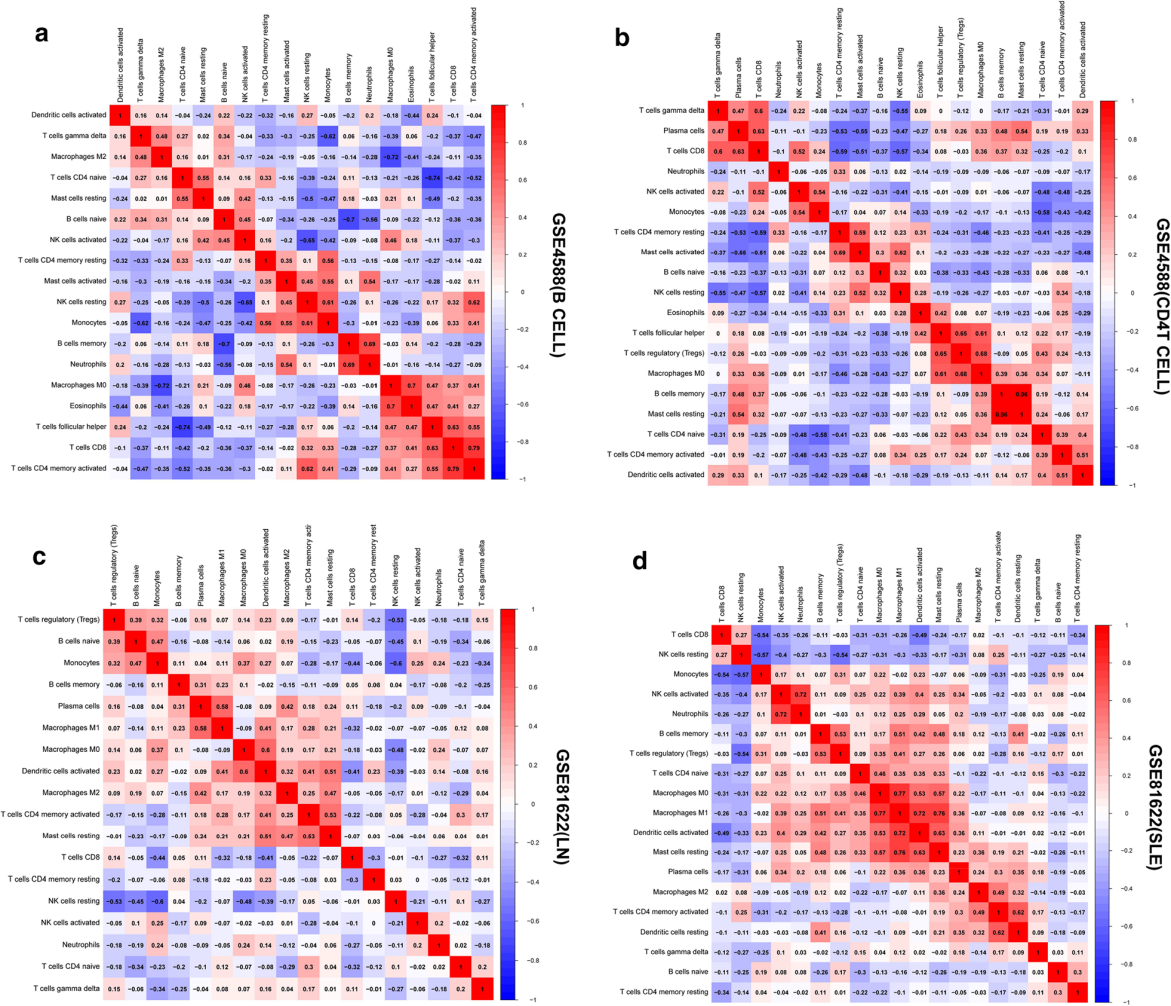
Correlation heat map of 22 types of immune cells. **a** GSE4588(B Cell), **b** GSE4588(CD4 T Cell), **c** GSE81622(LN), **d** GSE81622(SLE). The size of the colored squares represents the strength of the correlation. Red represents a positive correlation, blue represents a negative correlation. The darker the color, the stronger the correlation

**Fig. 11:**
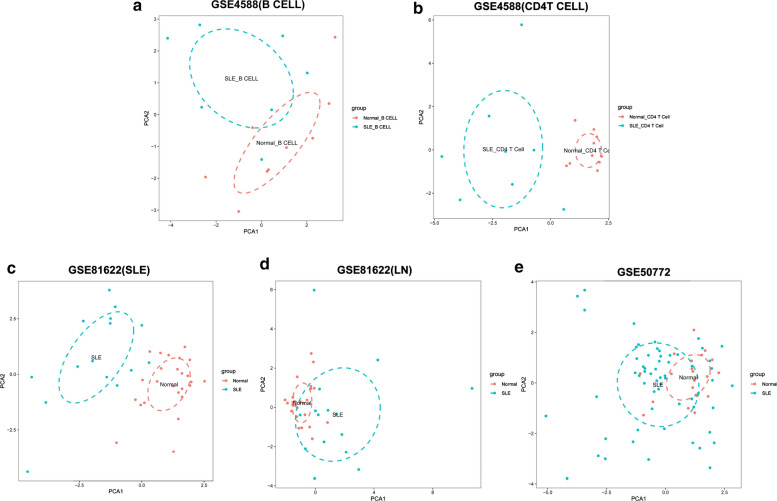
Principal components analyses performed on all samples of five datasets. The first two principal components which explain the most of the data variation are shown, **a** GSE4588(B Cell), **b** GSE4588(CD4 T Cell), **c** GSE81622(SLE), **d** GSE81622(LN), **e** GSE50772. This was indicative of the difference between the immune phenotypes of the groups

The violin plot of the immune cell infiltration difference showed that, compared with the normal control sample, T cells CD4 naïve (p = 0.055) in GSE4588 (CD4 T cell) infiltrated more, T cells CD4 memory resting (p = 0.001) in GSE4588 (CD4 T cell) infiltrated less (Fig. 8a). Moncytes (p = 0.014) and neutrophils (p < 0.001) in GSE50772 infiltrated more, NK cells resting (p < 0.001) and T cells CD4 memory resting (p < 0.001) in GSE50772 infiltrated less (Fig. 8b). GSE4588 (B cell) dataset have no significant immune cell infiltration showed in Additional file 17: Figure S5. Moncytes (p < 0.001) in GSE81622 (LN) infiltrated more, NK cells resting (p < 0.001) in GSE81622 (LN) infiltrated less (Fig. 9a). Moncytes (p < 0.001) in GSE81622 (SLE) infiltrated more, while NK cells resting (p < 0.001) in GSE81622 (SLE) infiltrated less (Fig. 9b). Correlation heatmap of the 22 types of immune cells revealed that B cells memory in GSE4588 (B cell) had a negative correlation with B cells naive (Fig. 10a). T cell CD8 in GSE4588 (CD4 T cell) had a significant positive correlation with T cell gamma delta, T cells follicular helper in GSE4588 (CD4 T cell) also had a positive correlation with T cells regulatory (Treg), T cells CD4 memory resting in GSE4588 (CD4 T cell) had a negative correlation with T cells CD8 (Fig. 10b). NK cells actived had a significant positive correlation with neutrophils in GSE81622 (SLE) (Fig. 10c). NK cells resting had a negative correlation with moncytes in GSE81622 (SLE) (Fig. 10c) and GSE81622 (LN) (Fig. 10d). In addition, We excluded dataset of GSE144390 because of the small number of samples, And GSE50772 dataset have no significant correlation of cell infiltration showed in Additional file 18: Figure S6. By PCA, the proportions of immune cells from the samples of SLE patients and normal controls displayed distinct group-bias clustering and individual differences. (Fig. 11a–e, Additional file 19: Table S14).

### Diagnose significance of DEGs

**Fig. 12:**
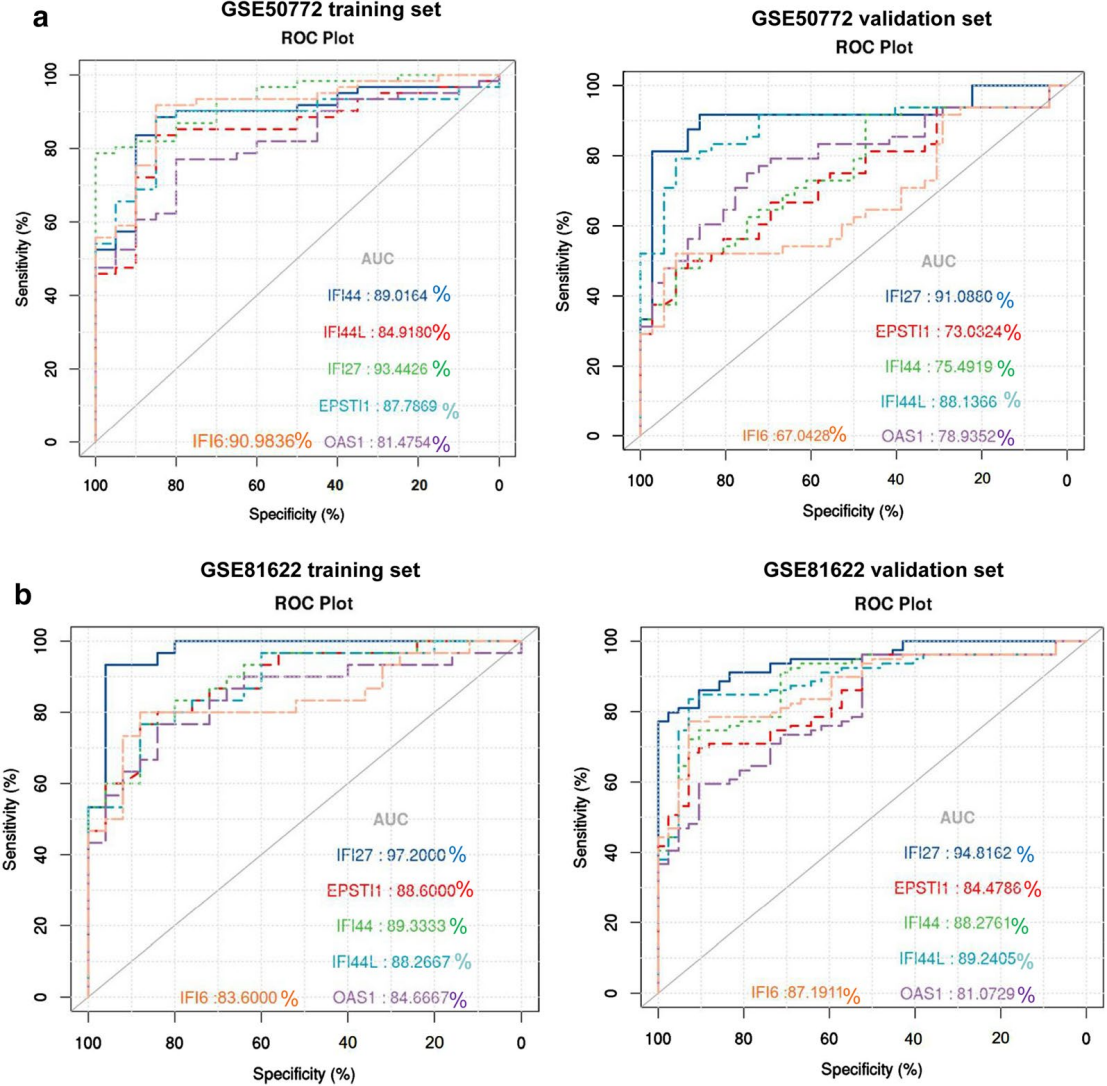
The diagnostic performance of the sixgenes. The diagnostic performance of the calculated based on the six genes expression in SLE diagnosis in training **a** GSE50772, **b** GSE81622 and two validation datasets, respectively. A AUC > 0.9 indicated that the model had a good fitting effect. ROC, receiver operating characteristic; AUC, area under the ROC curve

To determine which DEGs have the diagnose significance of SLE patients, The ROC analyses were conducted to explore the sensitivity and specificity of DEGs for SLE diagnosis. The results showed that IFI27 has the best diagnostic value for differentiating the patients with SLE from healthy controls (Fig. 12a, Additional file 20: Figure S7). The ROC curve analysis of the model in the GSE50772 (AUC = 0.934426), GSE81622 (AUC = 0.972000) training set demonstrated its promising predictive value for SLE. We then validated the model in the validation set, The AUC was 0.910880 and 0.948162 (Fig. 12a). This indicated that expression of IFI27 correlated with disease activity of SLE, IFI27 could act as a biomarker to estimate the activity of SLE and verify the effectiveness of the treatment of SLE.

## Discussion

SLE is one of the most common autoimmunity diseases worldwide [23]. Therefore, there is an urgent need for a better understanding of the detailed mechanism to develop novel strategies to diagnose and treat SLE [24].

In this study, a series of bioinformatics analysis identified 6 common DEGs (IFI27, IFI44, IFI44L, EPSTI1, OAS1) between SLE and normal samples based on gene expression profiles obtained from GSE50772, GSE81622(LN), GSE81622(SLE), GSE144390, GSE4588(B Cell), GSE4588(CD4 T Cell) and GSE144390 datasets. Furthermore, we investigated the biological functions of these common DEGs by using online website, and GO analysis revealed that these DEGs are significantly associated with changes in immune function and interferon response. Both pathways and GSEA enrichment analyses indicated that the interferon signaling pathway is a key pathway involved in SLE, which was in line with previous studies [25–29]. Moreover, the PPI network of DEGs was constructed by GENEMANIA. Those DEGs could contribute to promote the diagnostic and therapeutic in SLE, which could indicate a new direction of the acquaintance of SLE. To have a better understanding of the SLE progression, candidate biomarkers of SLE were identified using WGCNA in the current study. Finally, Some modules correlated with SLE were constructed by WGCNA analysis. IFI27 genes with high functional significance were selected as central genes in the clinical significance module. Then, We analyzed the correlation between these genes and patient diagnose. The ROC analyses were conducted to explore the sensitivity and specificity of DEGs for SLE diagnosis. Among the 6 common DEGs identified, IFI27 showed high sensitivity and specificity in SLE diagnosis in the training set (AUC > 0.9) and validation sets (AUC > 0.9). Thus, IFI27 may be a potential molecular signature for the diagnosis of SLE patients. Therefore, we speculate that IFI27 may play an important role in the disease progression of SLE. Since IFI27 exhibited the most dramatic difference in expression, we focused on the IFI27 gene in our subsequent experiments.

IFI27 (Interferon Alpha Inducible Protein 27), involved in different biological processes [30, 31]. Also involved in type-I interferon-induced apoptosis characterized by a rapid and robust release of cytochrome C from the mitochondria and activation of BAX and caspases 2, 3, 6, 8 and 9 [32]. In the innate immune response, IFI27 has an antiviral activity towards hepatitis C virus/HCV. May prevent the replication of the virus by recruiting both the hepatitis C virus non-structural protein 5A/NS5A and the ubiquitination machinery via SKP2, promoting the ubiquitin-mediated proteasomal degradation of NS5A [31, 33]. Although previous studies have explored the molecular mechanism by which diseases associated with IFI27 include Hepatitis C Virus and Oral Leukoplakia. Among its related pathways are Interferon gamma signaling and Innate Immune System [34, 35]. The relationship between IFI27 and the occurrence, progression of SLE has not been investigated. Other studies based on multiple datasets only focused on screening key genes [36–39], but did not specifcally analyse the molecular mechanism by which core genes play a role. Overall, our results indicate that targeting IFI27 might reduce the molecules that mediate immmune infection, suggesting that the potential value of combining blockades of IFI27 and coinhibitory molecules may serve as a new immunotherapy against SLE. In addition, research shows that immune cell infiltration plays an important role in the development of SLE [40]. Therefore, finding specific diagnostic markers and analyzing the pattern of SLE immune cell infiltration have profound significance for improving the prognosis of SLE patients. To further explore the role of immune cell infiltration in SLE, we used CIBERSORT to conduct a comprehensive evaluation of SLE immune infiltration. We found that an increased infiltration of moncytes, while NK cells resting infiltrated less may be related to the occurrence and development of SLE. Previous studies have shown that the infiltration of cells in the SLE is relatively high and is related to structural damage in patients with SLE [41]. It has also been shown that the tissue parenchyma has the capability of suppressing T cell responses and limiting damage to self. These findings suggest avenues for the treatment of autoimmunity based on selectively exploiting the exhausted phenotype of tissue-infiltrating T cells in SLE [42]. Hironari Hanaoka et al. [43] found that CD4 + Foxp3 + IL-17A + cells were infiltrated into the renal biopsy specimens of patients with active lupus nephritis. Liao et al. [44] confirmed through in vivo experiments that renal-infiltrating CD11c + cells are pathogenic in murine lupus nephritis through promoting CD4 + T cell responses. The above literature evidence combined with our analysis results have shown that IFI27 induced SLE among its related pathways are Interferon gamma signaling, And immune cell infiltration play important roles in SLE and should be the highlight of further studies.

SLE is a disease caused by the interaction of multiple susceptibility genes. In recent years, more and more genes have been found to be associated with SLE. Several potential candidate genes existenced in the MHC region of SLE, a study verified strong association of STAT4 gene rs7574865, rs10168266 polymorphisms and SLE susceptibility [45]. The PTPN22 rs1310182 A allele and rs1310182 AA genotype were associated with Pediatric systemic lupus erythematosus (PSLE) and may be a possible genetic marker for susceptibility to PSLE [46]. Different gene backgrounds lead to differences in the incidence of SLE. When the function of TREX1 is weakened, the abnormal accumulation of single-stranded DNA may stimulate the production of IFN, which may be one of the important factors contributing to the pathogenesis of SLE [47]. Sandling et al. [48] found that IKBKE and IL8 are susceptibility sites for SLE, emphasized the important function of the type I interferon pathway on the pathogenesis of SLE, but further analysis of the function remains to be seen. In addition to genetic factors, it is currently believed that the pathogenesis of SLE may be related to the abnormality of epigenetic modification. Yang et al. [49] identified five SLE related genes (CDKN1B, TET3, CD80, DRAM1 and ARID5B), revealing that cell cycle regulation, phagocytosis, DNA methylation and other mechanisms play an important role in the pathogenesis of SLE. This study also demonstrated that the pathogenesis of SLE has genetic heterogeneity. These candidate genes may play a pathogenic role through different biological pathways, and different gene mutations may lead to different system damage in SLE. The genetic pathogenesis of SLE will become a research hotspot once again.

In this study, we sought to identify biomarkers for SLE and further explore the role of immune cell infiltration in SLE. There are some limitations to our study. First, no further in vivo experiments to validate these results. Second, the exact mechanisms of immune reactions induced by IFI27 need to be further investigated. Third, CIBERSORT analysis is based on limited genetic data that may deviate from heterotypic interactions of cells, disease-induced disorders, or phenotypic plasticity. Terefore, our results still need to be verified through in vivo and in vitro experiments and clinical practice.

## Conclusion

In summary, based on integrated bioinformatical analyses, we identified differences in biological functions in SLE compared to normal samples and explored the comprehensive role of IFI27 in SLE progression. In particular, we found that IFI27 was positively correlated with immune function. To our knowledge, this is the first demonstration that IFI27 functions as a positive modulator in SLE. Thus, targeting IFI27 may have therapeutic promise for SLE. In addition, We found that an increased infiltration of moncytes, while NK cells resting infiltrated less may be related to the pathogenesis of SLE.

## Supplementary Information

**Additional file 1: Table S1.** Data cohort characteristics.

**Additional file 2: Table S2. **Data used for GSEA analysis.

**Additional file 3: Table S3. **Data used for WGCNA analysis.

**Additional file 4. **Scripts used for WGCNA.

**Additional file 5: Table S4. **Input data sets of immune cell infiltration.

**Additional file 6: Table S5. **Sample information of datasets.

**Additional file 7: Table S6. **Results of inmmune cell infiltration.

**Additional file 8. **Scripts used for immune cell infiltration.

**Additional file 9: Table S7. **DEGs in the datasets.

**Additional file 10: Figure S1. **Overlap between differently expressed gene lists of six datasets **Figure S2.** GO enrichment analyses of 6 DEGs from 6 datasets.

**Additional file 11: Table S8. **Pearson value between DEGs.

**Additional file 12: Table S9. **Pearson value between datasets.

**Additional file 13: Table S10.** miRNAs interact with circRNAs **Table S11. **miRNAs interact with lincRNAs.

**Additional file 14:** **Table S12.** miRNAs interact with mRNAs.

**Additional file 15: Figure S3. **Identification of weighted gene co-expression network modules associated with SLE in GSE4588(B cell) and GSE4588(CD4 T cell) datasets.** Figure S4. **Identification of weighted gene co-expression network modules associated with SLE in GSE81622 and GSE50772 datasets.

**Additional file 16: Table S13. **Co-expression modules results of datasets.

**Additional file 17: Figure S5.** Violin diagram of the proportion of 22 types of immune cells in GSE4588 (B cell) dataset.

**Additional file 18: Figure S6. ** Cell infiltration of the GSE50772 dataset.

**Additional file 19: Table S14.** PCA results of immune cell infiltration.

**Additional file 20: Figure S7.** The diagnostic performance of the six genes of three datasets.

## Data Availability

The datasets generated during and/or analyzed during the current study are available in the Gene Expression Omnibus (GEO) datasets (http://www.ncbi.nlm.nih.gov/geo/).

## Abbreviations

SLE: Systemic lupus erythematosus
DEGs: Differentially expressed genes
GO: Gene ontology
KEGG: Kyoto Encyclopedia of Genes and Genomes
PPI: Protein–protein interaction
GSEA: Gene set enrichment analysis
WGCNA: Weighted gene co‐expression network analysis
IFI27: Interferon Alpha Inducible Protein 27
PCA: Principal component analysis
ROC: Receiver operating characteristic
TOM: Topological overlap matrix
GEO: Gene expression omnibus
miRNA: MicroRNA
LncRNA: Long non-coding RNA
PBMC: Peripheral blood mononuclear cell
FC: Fold-change
IFI44: Interferon induced protein 44
IFI44L: Interferon induced protein 44 like
IFI6: Interferon induced protein 6
OAS1: 2′-5′-Oligoadenylate Synthetase 1
EPSTI1: Epithelial Stromal Interaction 1
TNFSF4: TNF Superfamily Member 4
NCF1: Neutrophil Cytosolic Factor 1
CXorf21: Chromosome X Open Reading Frame 21
IFIT1: Interferon Induced Protein With Tetratricopeptide Repeats 1
IFIT3: Interferon Induced Protein With Tetratricopeptide Repeats 3
GMP: Guanine monophosphate
AMP: Adenosine monophosphate
cGAS: Cyclic GMP-AMP synthase
NS5A: Non-structural protein 5A
SKP2: S-Phase Kinase Associated Protein 2
BAX: BCL2 Associated X, Apoptosis Regulator
HCV: Hepatitis C virus
STAT4: Signal Transducer And Activator Of Transcription 4
PTPN22: Protein Tyrosine Phosphatase Non-Receptor Type 22
PSLE: Pediatric systemic lupus erythematosus
TREX1: Three Prime Repair Exonuclease 1
IL8: Interleukin 8
IKBKE: Inhibitor Of Nuclear Factor Kappa B Kinase Subunit Epsilon
CDKN1B: Cyclin Dependent Kinase Inhibitor 1B
TET3: Tet Methylcytosine Dioxygenase 3
CD80: CD80 Molecule
DRAM1: DNA Damage Regulated Autophagy Modulator 1
ARID5B: AT-Rich Interaction Domain 5B
