# Correction to: Identification of key biomarkers and immune infiltration in systemic lupus erythematosus by integrated bioinformatics analysis

**DOI:** 10.1186/s12967-021-02728-2

**Published:** 2021-02-11

**Authors:** Xingwang Zhao, Longlong Zhang, Juan Wang, Min Zhang, Zhiqiang Song, Bing Ni, Yi You

**Affiliations:** 1 Department of Dermatology, Southwest Hospital, Army Medical University, Third Military Medical University, Chongqing, 400038 China; 2 Department of Pathophysiology, College of High Altitude Military Medicine, Army Medical University, Third Military Medical University, Chongqing, China; 3 State Key Laboratory of Genetic Resources and Evolution, Kunming Institute of Zoology, Chinese Academy of Sciences, Kunming, 650223 China; 4 Kunming College of Life Science, University of Chinese Academy of Sciences, Kunming, 650204 China

## Correction to: J Transl Med (2021) 19:35 10.1186/s12967-020-02698-x

Following publication of the original article [1], the authors identified an error in the corresponding authorship. In the original article, Bing Ni is indicated as the sole corresponding author. However, Bing Ni shares corresponding authorship with Yi You.

The corrected corresponding authorship been updated in the author group above and the original article [1] has been corrected. The publisher apologises to the authors and readers for the inconvenience caused by this error.
