# Supplementary material for: Identification of key biomarkers and immune infiltration in systemic lupus erythematosus by integrated bioinformatics analysis
Source: J Transl Med. 2021 Jan 19;19:35. doi: 10.1186/s12967-020-02698-x (PMC7814551; doi:10.1186/s12967-020-02698-x)
Supplement: Supplementary file 1 — Additional file 1: Table S1. Data cohort characteristics. [file 12967_2020_2698_MOESM1_ESM.doc]

**Additional file 1: Table S1. Data cohort characteristics**

| **Data set** | **Use** | **SLE (N)** | **Normal (N)** |
| --- | --- | --- | --- |
| **GSE4588(CD4 T cells)** | **Integration Analysis** | **8** | **10** |
| **GSE4588(B cells)** | **Integration Analysis** | **7** | **9** |
| **GSE81622(SLE)** | **Integration Analysis** | **15** | **25** |
| **GSE81622 (LN)** | **Integration Analysis** | **15** | **25** |
| **GSE144390** | **Integration Analysis** | **3** | **3** |
| **GSE50772** | **Integration Analysis** | **61** | **20** |

**SLE: Systemic Lupus Erythematosus.**
