# Supplementary material for: Identification of key biomarkers and immune infiltration in systemic lupus erythematosus by integrated bioinformatics analysis
Source: J Transl Med. 2021 Jan 19;19:35. doi: 10.1186/s12967-020-02698-x (PMC7814551; doi:10.1186/s12967-020-02698-x)

**Additional file 10: Figure S1. Overlap between differently expressed gene lists of six datasets**


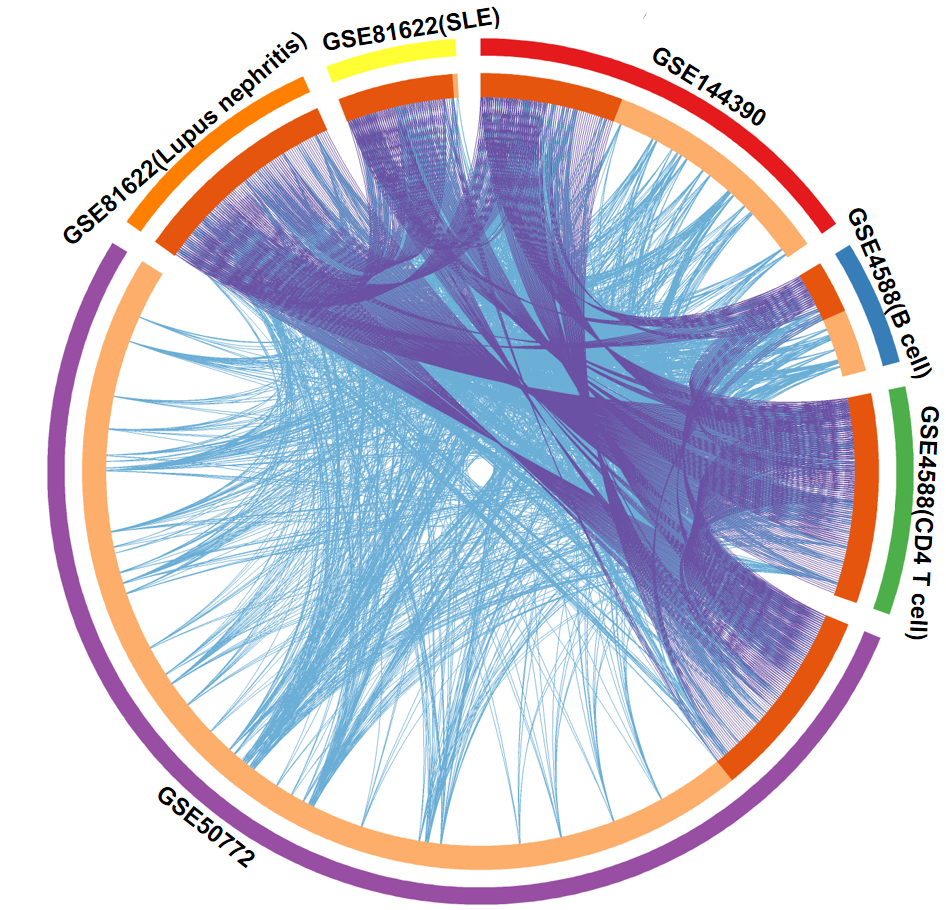


**Supplementary Figure S2: GO enrichment analyses of 6 DEGs from the six datasets.**


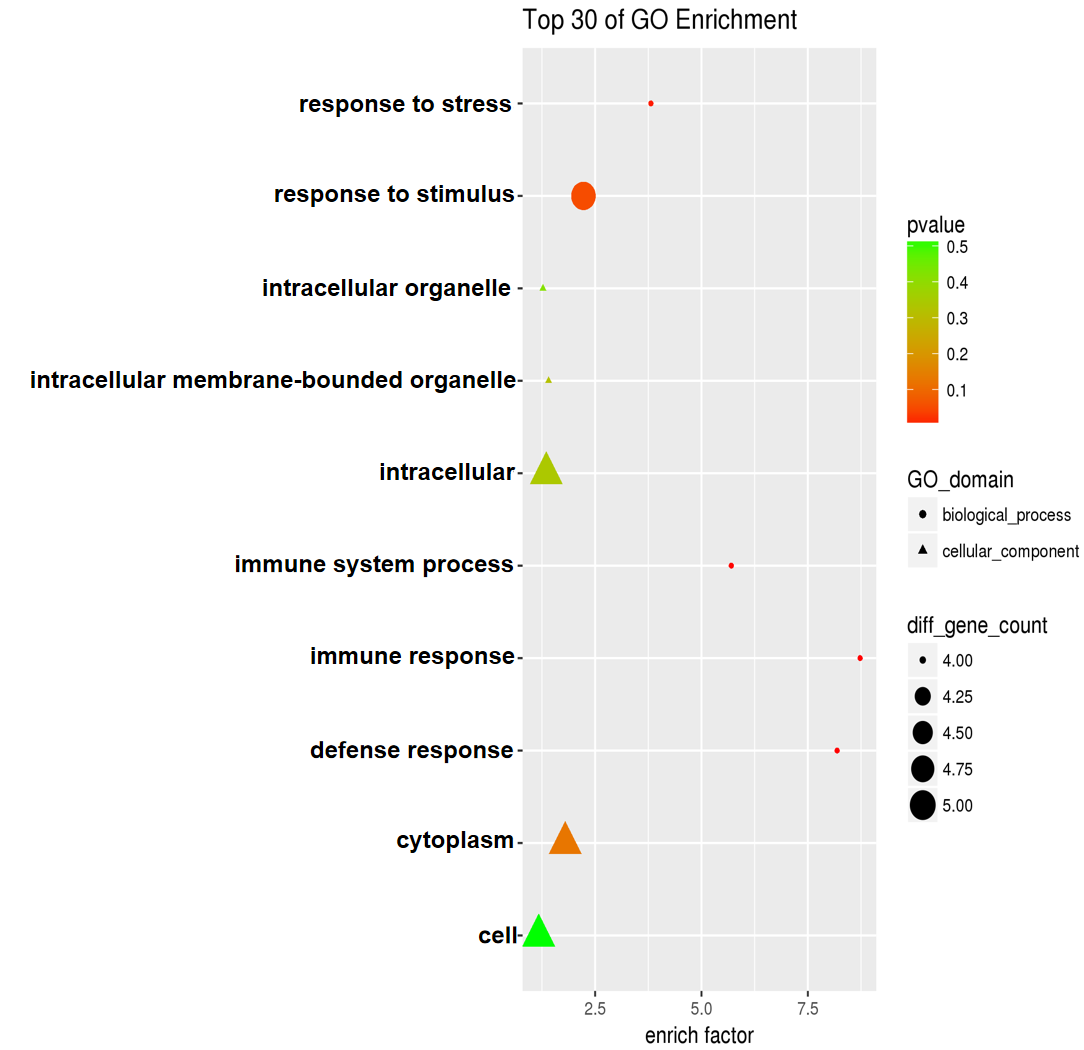

Supplement: Supplementary file 10 — Additional file 10: Figure S1. Overlap between differently expressed gene lists of six datasets Figure S2. GO enrichment analyses of 6 DEGs from 6 datasets. [file 12967_2020_2698_MOESM10_ESM.doc]
