# Supplementary material for: Identification of key biomarkers and immune infiltration in systemic lupus erythematosus by integrated bioinformatics analysis
Source: J Transl Med. 2021 Jan 19;19:35. doi: 10.1186/s12967-020-02698-x (PMC7814551; doi:10.1186/s12967-020-02698-x)
Supplement: Supplementary file 13 — Additional file 13: Table S10. miRNAs interact with circRNAs Table S11. miRNAs interact with lincRNAs. [file 12967_2020_2698_MOESM13_ESM.doc]

**Additional file 13: Table S10. miRNAs interact with circRNAs**

| **miRNAname** | **geneName** | **geneType** | **chromosome** | **start** | **end** | **clipExpNum** |
| --- | --- | --- | --- | --- | --- | --- |
| **hsa-miR-34a-5p** | **LDHA** | **circRNA** | **chr11** | **18429069** | **18429092** | **32** |
| **hsa-miR-34a-5p** | **DICER1** | **circRNA** | **chr14** | **95571486** | **95571507** | **36** |
| **hsa-miR-34a-5p** | **XBP1** | **circRNA** | **chr22** | **29191317** | **29191337** | **31** |
| **hsa-miR-34c-5p** | **LDHA** | **circRNA** | **chr11** | **18429069** | **18429092** | **32** |
| **hsa-miR-34c-5p** | **DICER1** | **circRNA** | **chr14** | **95571486** | **95571507** | **36** |
| **hsa-miR-34c-5p** | **XBP1** | **circRNA** | **chr22** | **29191317** | **29191337** | **31** |
| **hsa-miR-485-5p** | **HSP90AB1** | **circRNA** | **chr6** | **44221384** | **44221404** | **36** |
| **hsa-miR-485-5p** | **ABCF2** | **circRNA** | **chr7** | **150911013** | **150911034** | **33** |
| **hsa-miR-485-5p** | **ACTG1** | **circRNA** | **chr17** | **79477367** | **79477391** | **35** |
| **hsa-miR-485-5p** | **ZNF460** | **circRNA** | **chr19** | **57805145** | **57805166** | **34** |
| **hsa-miR-485-5p** | **DDX17** | **circRNA** | **chr22** | **38882028** | **38882049** | **39** |
| **hsa-miR-589-5p** | **INSIG1** | **circRNA** | **chr7** | **155101073** | **155101094** | **32** |
| **hsa-miR-589-5p** | **SET** | **circRNA** | **chr9** | **131457075** | **131457096** | **32** |
| **hsa-miR-589-5p** | **RPL17-C18ORF32** | **circRNA** | **chr18** | **47008392** | **47008413** | **40** |
| **hsa-miR-449b-5p** | **LDHA** | **circRNA** | **chr11** | **18429071** | **18429092** | **32** |
| **hsa-miR-449b-5p** | **DICER1** | **circRNA** | **chr14** | **95571486** | **95571508** | **36** |
| **hsa-miR-449b-5p** | **XBP1** | **circRNA** | **chr22** | **29191317** | **29191338** | **31** |
| **hsa-miR-660-5p** | **CSDE1** | **circRNA** | **chr1** | **115282465** | **115282484** | **36** |
| **hsa-miR-660-5p** | **PRSS23** | **circRNA** | **chr11** | **85195179** | **85195190** | **45** |
| **hsa-miR-660-5p** | **ZFYVE26** | **circRNA** | **chr14** | **68213475** | **68213494** | **37** |
| **hsa-miR-660-5p** | **APP** | **circRNA** | **chr21** | **27253743** | **27253763** | **36** |
| **hsa-miR-186-5p** | **TNPO1** | **circRNA** | **chr5** | **72168520** | **72168541** | **39** |
| **hsa-miR-186-5p** | **TMBIM6** | **circRNA** | **chr12** | **50158617** | **50158635** | | **66** | | --- | |
| **hsa-miR-186-5p** | **FAM108C1** | **circRNA** | **chr15** | **81047847** | **81047865** | **31** |
| **hsa-miR-9-5p** | **CTNNB1** | **circRNA** | **chr3** | **41275087** | **41275108** | **44** |
| **hsa-miR-9-5p** | **RPLP0** | **circRNA** | **chr12** | **120638638** | **120638659** | **38** |
| **hsa-miR-9-5p** | **GARS** | **circRNA** | **chr7** | **30665891** | **30665912** | **33** |
| **hsa-miR-9-5p** | **MYC** | **circRNA** | **chr8** | **128751143** | **128751162** | **35** |
| **hsa-miR-9-5p** | **KPNB1** | **circRNA** | **chr17** | **45735988** | **45736010** | **28** |
| **hsa-miR-522-3p** | **TNPO1** | **circRNA** | **chr5** | **72206445** | **72206464** | **30** |
| **hsa-miR-522-3p** | **TMBIM6** | **circRNA** | **chr12** | **50158617** | **50158635** | | **66** | | --- | |
| **hsa-miR-522-3p** | **FAM108C1** | **circRNA** | **chr15** | **81047847** | **81047865** | **31** |
| **hsa-miR-522-3p** | **PFN1** | **circRNA** | **chr17** | **4849118** | **4849139** | **37** |
| **hsa-miR-522-3p** | **CAPNS1** | **circRNA** | **chr19** | **36636900** | **36636921** | **39** |
| **hsa-miR-186-5p** | **CTNNB1** | **circRNA** | **chr5** | **72206445** | **72206464** | **30** |
| **hsa-miR-185-5p** | **BTG2** | **circRNA** | **chr1** | **203277701** | **203277719** | **36** |
| **hsa-miR-185-5p** | **TNRC6A** | **circRNA** | **chr16** | **24835523** | **24835543** | **46** |
| **hsa-miR-182-5p** | **ADSS** | **circRNA** | **chr1** | **244572678** | **244572701** | **34** |
| **hsa-miR-182-5p** | **NUP43** | **circRNA** | **chr6** | **150047130** | **150047153** | **43** |
| **hsa-miR-182-5p** | **KLHL28** | **circRNA** | **chr14** | **45397990** | **45398013** | **33** |
| **hsa-miR-182-5p** | **FTL** | **circRNA** | **chr19** | **49468737** | **49468759** | **31** |
| **hsa-miR-182-5p** | **XBP1** | **circRNA** | **chr22** | **29191193** | **29191215** | **34** |
| **hsa-miR-27a-3p** | **TMBIM6** | **circRNA** | **chr12** | **50158617** | **50158635** | | **66** | | --- | |
| **hsa-miR-27a-3p** | **TNPO1** | **circRNA** | **chr5** | **72206174** | **72206194** | **47** |
| **hsa-miR-27b-3p** | **TNPO1** | **circRNA** | **chr5** | **72206174** | **72206194** | **47** |
| **hsa-miR-27b-3p** | **TMBIM6** | **circRNA** | **chr12** | **50158617** | **50158635** | **66** |

**Additional file 12-Supplementary Table S11 miRNAs interact with lincRNAs**

| **miRNAname** | **geneName** | **geneType** | **chromosome** | **start** | **end** | **clipExpNum** |
| --- | --- | --- | --- | --- | --- | --- |
| **hsa-miR-34a-5p** | **SNHG7** | **lincRNA** | **chr9** | **139621325** | **139621345** | **16** |
| **hsa-miR-34a-5p** | **NEAT1** | **lincRNA** | **chr11** | **65205208** | **65205230** | **19** |
| **hsa-miR-34c-5p** | **SNHG7** | **lincRNA** | **chr9** | **139621325** | **139621348** | **16** |
| **hsa-miR-34c-5p** | **NEAT1** | **lincRNA** | **chr11** | **65205207** | **65205230** | **19** |
| **hsa-miR-34c-5p** | **AC116913.1** | **lincRNA** | **chr15** | **66782460** | **66782482** | **11** |
| **hsa-miR-34c-5p** | **AC018628.1** | **lincRNA** | **chr17** | **60081737** | **60081758** | **15** |
| **hsa-miR-485-5p** | **MIR29B2CHG** | **lincRNA** | **chr1** | **207975182** | **207975203** | **13** |
| **hsa-miR-485-5p** | **AC074117.1** | **lincRNA** | **chr2** | **27580726** | **27580748** | **12** |
| **hsa-miR-485-5p** | **NEAT1** | **lincRNA** | **chr11** | **65198512** | **65198534** | **15** |
| **hsa-miR-485-5p** | **MALAT1** | **lincRNA** | **chr11** | **65272750** | **65272771** | **30** |
| **hsa-miR-485-5p** | **AC016876.2** | **lincRNA** | **chr17** | **7482121** | **7482140** | **13** |
| **hsa-miR-485-5p** | **AC005899.4** | **lincRNA** | **chr17** | **30687656** | **30687681** | **24** |
| **hsa-miR-589-5p** | **MALAT1** | **lincRNA** | **chr11** | **65269721** | **65269743** | **26** |
| **hsa-miR-589-5p** | **AC016876.2** | **lincRNA** | **chr17** | **7480009** | **7480029** | **10** |
| **hsa-miR-589-5p** | **AC024267.1** | **lincRNA** | **chr17** | **27188400** | **27188422** | **25** |
| **hsa-miR-449b-5p** | **SNHG7** | **lincRNA** | **chr9** | **139621325** | **139621346** | **16** |
| **hsa-miR-449b-5p** | **NEAT1** | **lincRNA** | **chr11** | **65205207** | **65205230** | **19** |
| **hsa-miR-660-5p** | **LINC00641** | **lincRNA** | **chr14** | **21670292** | **21670313** | **18** |
| **hsa-miR-660-5p** | **SNHG16** | **lincRNA** | **chr17** | **74559581** | **74559603** | **16** |
| **hsa-miR-27a-3p** | **AC010980.2** | **lincRNA** | **chr2** | **223182577** | **223182595** | **18** |
| **hsa-miR-27a-3p** | **NEAT1** | **lincRNA** | **chr11** | **65192982** | **65193001** | **17** |
| **hsa-miR-1294** | **AC007161.3** | **lincRNA** | **chr7** | **8043604** | **8043626** | **13** |
| **hsa-miR-522-3p** | **MIR29B2CHG** | **lincRNA** | **chr1** | **207975220** | **207975241** | **43** |
| **hsa-miR-522-3p** | **AC016831.1** | **lincRNA** | **chr7** | **130562237** | **130562258** | **38** |
| **hsa-miR-522-3p** | **NEAT1** | **lincRNA** | **chr11** | **65191624** | **65191645** | **17** |
| **hsa-miR-186-5p** | **SNHG3** | **lincRNA** | **chr1** | **28835694** | **28835715** | **16** |
| **hsa-miR-186-5p** | **AC010435.1** | **lincRNA** | **chr5** | **43586539** | **43586561** | **11** |
| **hsa-miR-186-5p** | **AC124798.1** | **lincRNA** | **chr11** | **17402461** | **17402482** | **15** |
| **hsa-miR-186-5p** | **AC134407.3** | **lincRNA** | **chr17** | **65972022** | **65972043** | **11** |
| **hsa-miR-9-5p** | **AC023509.1** | **lincRNA** | **chr12** | **53848522** | **53848543** | **19** |
| **hsa-miR-9-5p** | **TUG1** | **lincRNA** | **chr22** | **31373316** | **31373339** | **16** |
| **hsa-miR-9-5p** | **XIST** | **lincRNA** | **chrX** | **73042802** | **73042824** | **18** |
| **hsa-miR-185-5p** | **MALAT1** | **lincRNA** | **chr11** | **65272241** | **65272262** | **26** |
| **hsa-miR-185-5p** | **AC007952.4** | **lincRNA** | **chr17** | **19015772** | **19015792** | **34** |
| **hsa-miR-182-5p** | **SNHG1** | **lincRNA** | **chr11** | **62619696** | **62619719** | **17** |
| **hsa-miR-182-5p** | **AL137129.1** | **lincRNA** | **chr14** | **62200880** | **62200905** | **19** |
| **hsa-miR-182-5p** | **NORAD** | **lincRNA** | **chr20** | **34636165** | **34636188** | **18** |
| **hsa-miR-27b-3p** | **AC010980.2** | **lincRNA** | **chr2** | **223182577** | **223182595** | **18** |
| **hsa-miR-27b-3p** | **NEAT1** | **lincRNA** | **chr11** | **65192982** | **65193001** | **17** |
