# Supplementary material for: Identification of key biomarkers and immune infiltration in systemic lupus erythematosus by integrated bioinformatics analysis
Source: J Transl Med. 2021 Jan 19;19:35. doi: 10.1186/s12967-020-02698-x (PMC7814551; doi:10.1186/s12967-020-02698-x)
Supplement: Supplementary file 14 — Additional file 14: Table S12. miRNAs interact with mRNAs. [file 12967_2020_2698_MOESM14_ESM.doc]

**Additional file 14: Table S11 miRNAs interact with mRNAs**

| **geneName** | **miRNAname** | **PITA** | **RNA22** | **miRmap** | **microT** | **miRanda** | **PicTar** | **TargetScan** |
| --- | --- | --- | --- | --- | --- | --- | --- | --- |
| **IFI6** | **hsa-miR-589-5p** | **1** | **0** | **1** | **1** | **0** | **0** | **0** |
| **IFI6** | **hsa-miR-485-5p** | **1** | **1** | **1** | **0** | **1** | **0** | **0** |
| **EPSTI1** | **hsa-miR-34a-5p** | **1** | **0** | **1** | **1** | **0** | **0** | **0** |
| **EPSTI1** | **hsa-miR-34c-5p** | **1** | **0** | **1** | **1** | **1** | **0** | **0** |
| **EPSTI1** | **hsa-miR-449b-5p** | **1** | **0** | **1** | **1** | **0** | **0** | **0** |
| **EPSTI1** | **hsa-miR-660-5p** | **1** | **0** | **1** | **1** | **0** | **0** | **0** |
| **IFI27** | **hsa-miR-182-5p** | **1** | **0** | **0** | **1** | **0** | **1** | **0** |
| **IFI44** | **hsa-miR-27a-3p** | **1** | **0** | **1** | **0** | **1** | **0** | **0** |
| **IFI44** | **hsa-miR-1294** | **1** | **0** | **1** | **0** | **0** | **0** | **0** |
| **IFI44** | **hsa-miR-27b-3p** | **1** | **0** | **0** | **0** | **1** | **0** | **0** |
| **IFI44L** | **hsa-miR-185-5p** | **1** | **0** | **1** | **0** | **1** | **0** | **0** |
| **OAS1** | **hsa-miR-9-5p** | **1** | **0** | **1** | **1** | **0** | **0** | **1** |
| **OAS1** | **hsa-miR-186-5p** | **1** | **0** | **1** | **0** | **1** | **0** | **0** |
| **OAS1** | **hsa-miR-522-3p** | **1** | **0** | **1** | **1** | **0** | **0** | **0** |
