# Supplementary material for: Identification of key biomarkers and immune infiltration in systemic lupus erythematosus by integrated bioinformatics analysis
Source: J Transl Med. 2021 Jan 19;19:35. doi: 10.1186/s12967-020-02698-x (PMC7814551; doi:10.1186/s12967-020-02698-x)

**Additional file 15: Figure S3. Identification of weighted gene co-expression network modules associated with SLE in two datasets.**


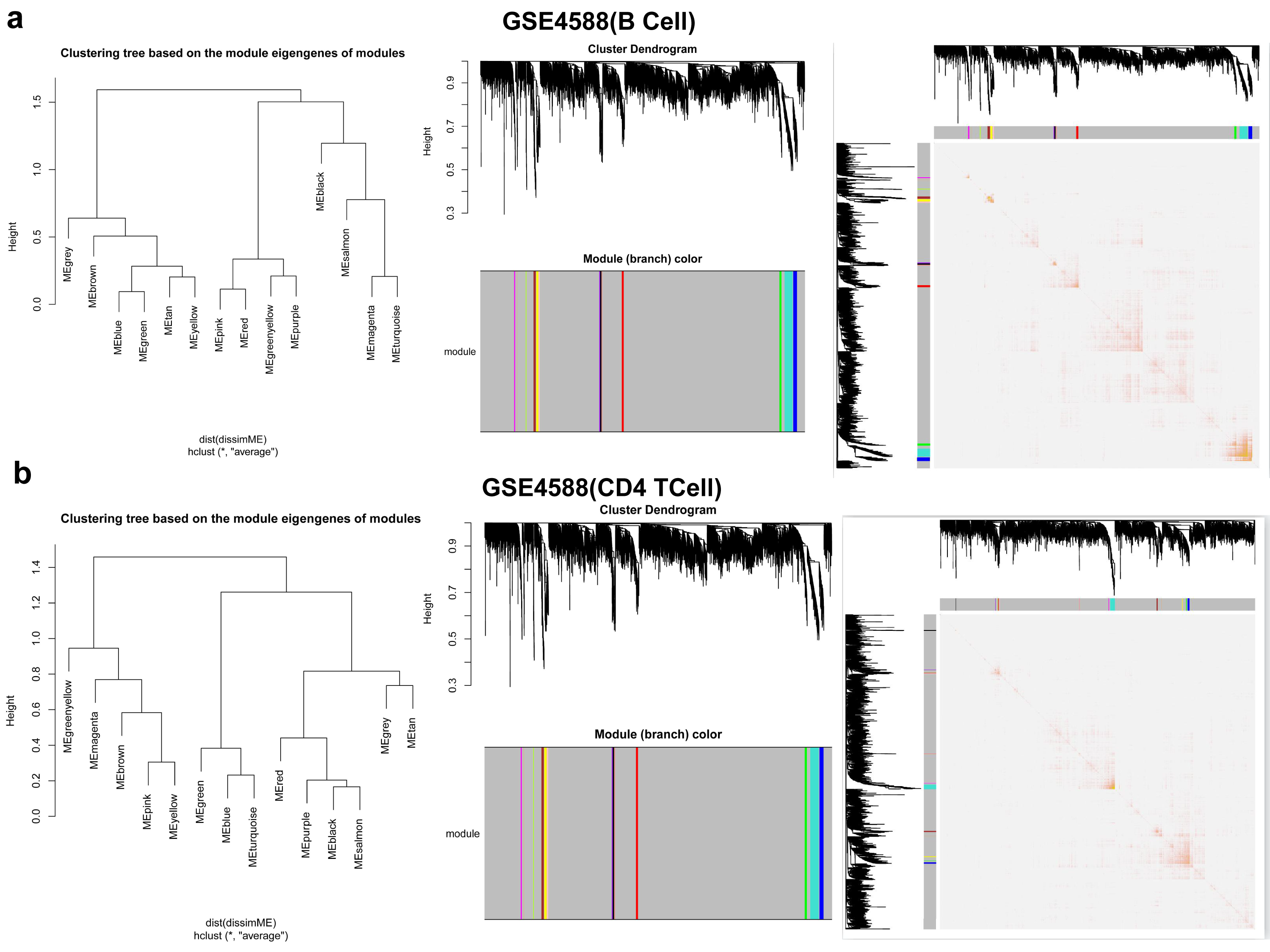


**Supplementary Figure S4: Identification of weighted gene co-expression network modules associated with SLE in two datasets.**


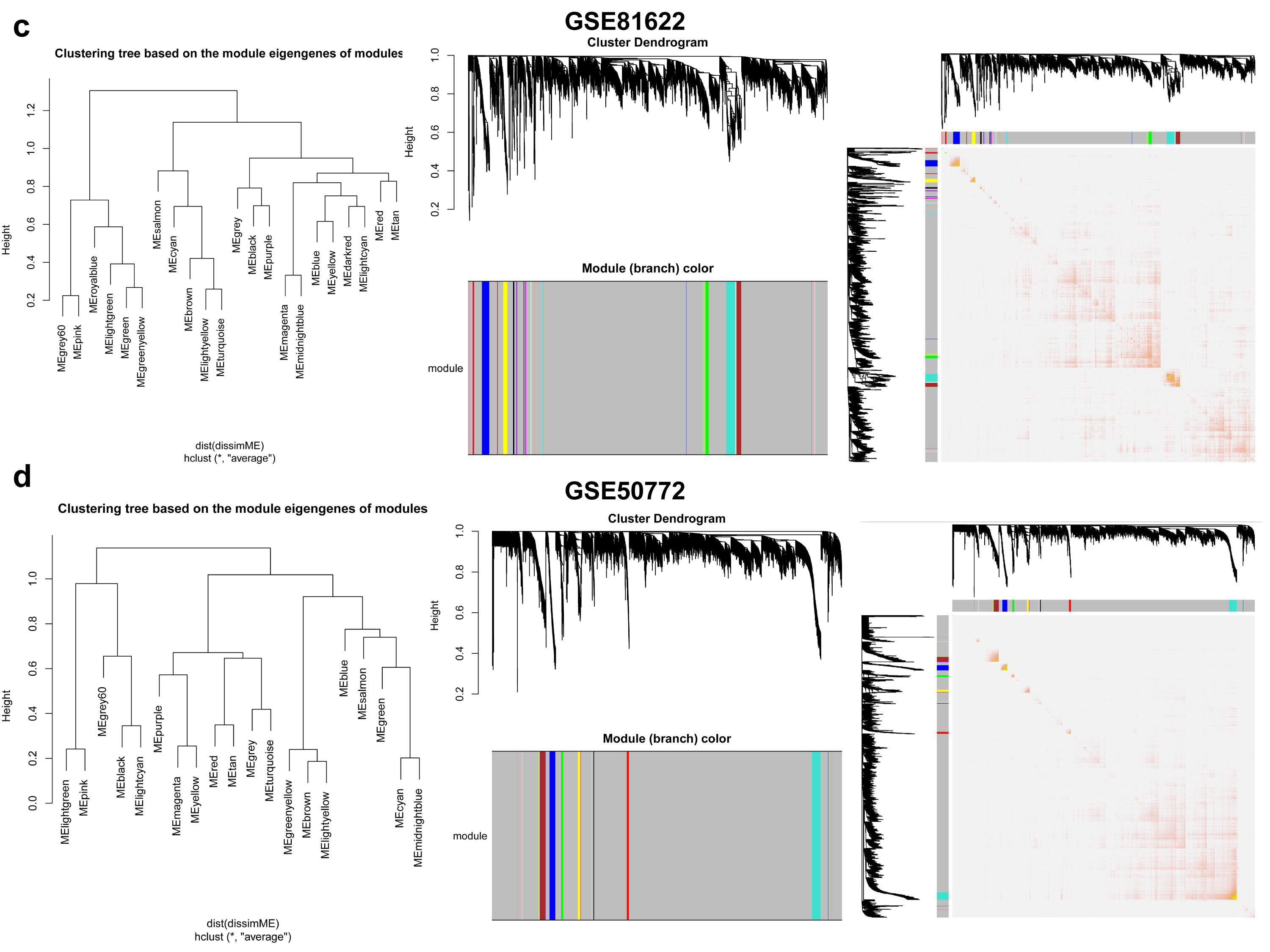

Supplement: Supplementary file 15 — Additional file 15: Figure S3. Identification of weighted gene co-expression network modules associated with SLE in GSE4588(B cell) and GSE4588(CD4 T cell) datasets. Figure S4. Identification of weighted gene co-expression network modules associated with SLE in GSE81622 and GSE50772 datasets. [file 12967_2020_2698_MOESM15_ESM.doc]
