# Supplementary material for: Identification of key biomarkers and immune infiltration in systemic lupus erythematosus by integrated bioinformatics analysis
Source: J Transl Med. 2021 Jan 19;19:35. doi: 10.1186/s12967-020-02698-x (PMC7814551; doi:10.1186/s12967-020-02698-x)
Supplement: Supplementary file 17 — Additional file 17: Figure S5. Violin diagram of the proportion of 22 types of immune cells in GSE4588 (B cell) dataset. [file 12967_2020_2698_MOESM17_ESM.doc]

**Additional file 17: Figure S5. Violin diagram of the proportion of 22 types of immune cells in GSE4588 (B cell) dataset.**

**
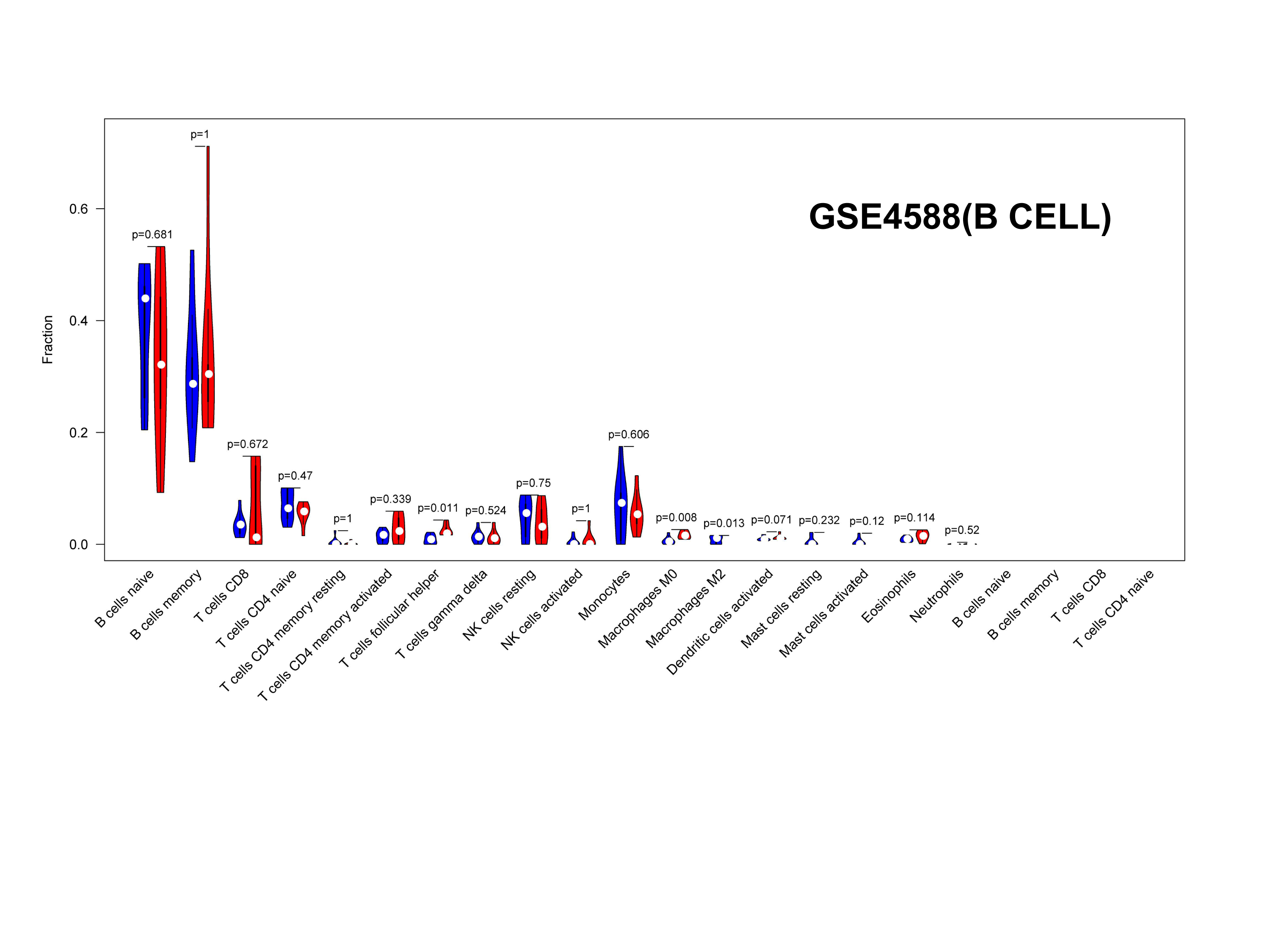
**
