# Supplementary figures and images for: Identification of key biomarkers and immune infiltration in systemic lupus erythematosus by integrated bioinformatics analysis
Source: J Transl Med. 2021 Jan 19;19:35. doi: 10.1186/s12967-020-02698-x (PMC7814551; doi:10.1186/s12967-020-02698-x)

**Additional file 18: Figure S6. cell infiltration of the GSE50772 dataset .**


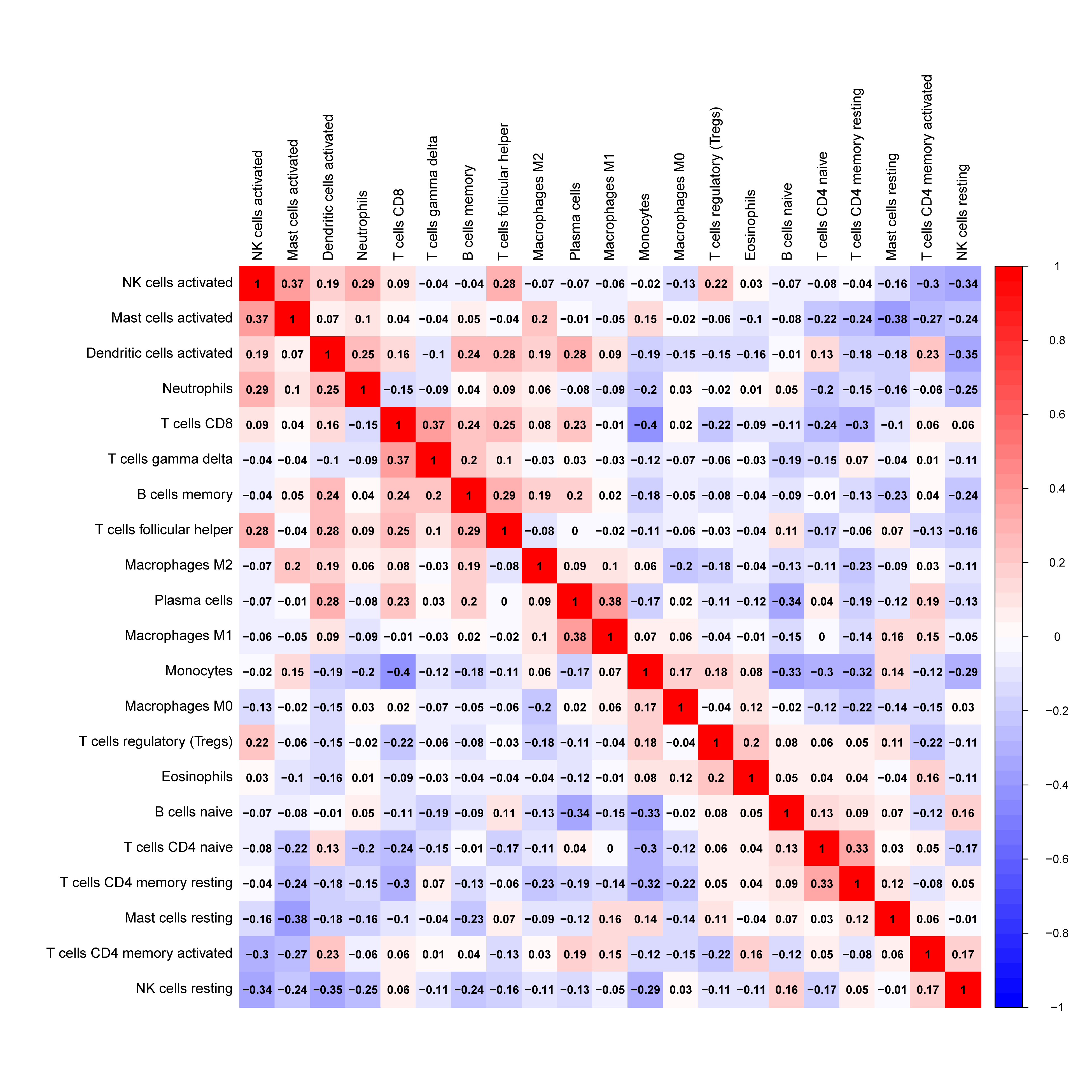

Supplement: Supplementary file 18 — Additional file 18: Figure S6. Cell infiltration of the GSE50772 dataset. [file 12967_2020_2698_MOESM18_ESM.doc]

**Additional file 20: Figure S7. The diagnostic performance of the six genes of three datasets.**


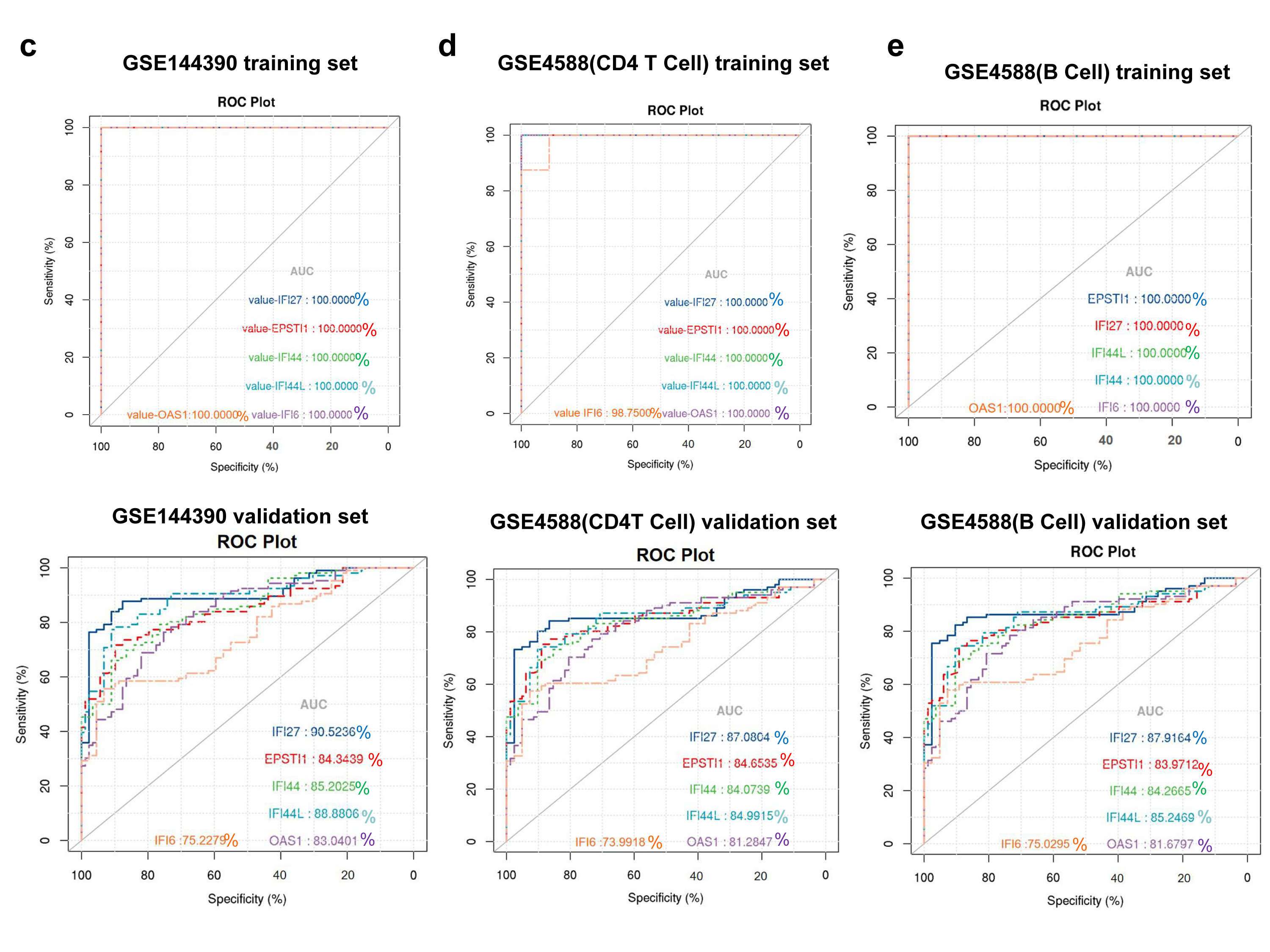

Supplement: Supplementary file 20 — Additional file 20: Figure S7. The diagnostic performance of the six genes of three datasets. [file 12967_2020_2698_MOESM20_ESM.doc]
